# Insulin/IGF-1 Drives PERIOD Synthesis to Entrain Circadian Rhythms with Feeding Time

**DOI:** 10.1016/j.cell.2019.02.017

**Published:** 2019-05-02

**Authors:** Priya Crosby, Ryan Hamnett, Marrit Putker, Nathaniel P. Hoyle, Martin Reed, Carolyn J. Karam, Elizabeth S. Maywood, Alessandra Stangherlin, Johanna E. Chesham, Edward A. Hayter, Lyn Rosenbrier-Ribeiro, Peter Newham, Hans Clevers, David A. Bechtold, John S. O’Neill

**Affiliations:** 1 MRC Laboratory of Molecular Biology, Cambridge CB2 0QH, UK; 2 Hubrecht Institute, Utrecht 3584 CT, the Netherlands; 3 Drug Safety and Metabolism, IMED Biotech Unit, AstraZeneca, Cambridge CB4 0FZ, UK; 4 Princess Máxima Centre, Utrecht 3584 CS, the Netherlands; 5 Faculty of Biology, Medicine and Health, University of Manchester, Manchester M13 9PL, UK

**Keywords:** circadian, food entrainment, insulin, IGF-1, PERIOD, mTORC1, miRNA

## Abstract

In mammals, endogenous circadian clocks sense and respond to daily feeding and lighting cues, adjusting internal ∼24 h rhythms to resonate with, and anticipate, external cycles of day and night. The mechanism underlying circadian entrainment to feeding time is critical for understanding why mistimed feeding, as occurs during shift work, disrupts circadian physiology, a state that is associated with increased incidence of chronic diseases such as type 2 (T2) diabetes. We show that feeding-regulated hormones insulin and insulin-like growth factor 1 (IGF-1) reset circadian clocks *in vivo* and *in vitro* by induction of PERIOD proteins, and mistimed insulin signaling disrupts circadian organization of mouse behavior and clock gene expression. Insulin and IGF-1 receptor signaling is sufficient to determine essential circadian parameters, principally *via* increased PERIOD protein synthesis. This requires coincident mechanistic target of rapamycin (mTOR) activation, increased phosphoinositide signaling, and microRNA downregulation. Besides its well-known homeostatic functions, we propose insulin and IGF-1 are primary signals of feeding time to cellular clocks throughout the body.

## Highlights

• Insulin and IGF-1 are a systemic synchronizing cue for circadian rhythms in mammals
• Insulin and IGF-1 signaling rapidly upregulates translation of PERIOD clock proteins
• Coincident signaling facilitates selective induction of PERIOD synthesis
• Circadian disruption is recapitulated by mistimed insulin in cell and animal models

## Graphical Abstract

**Figure:**
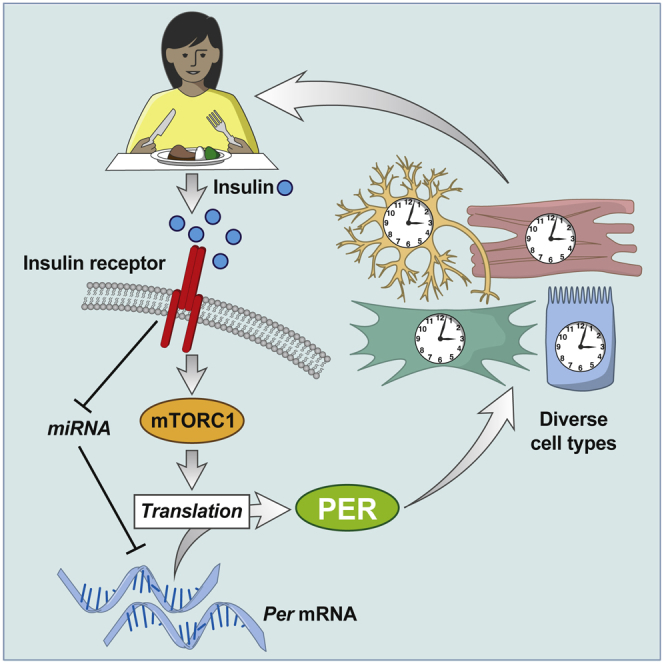

## Teaser

Feeding-associated hormones insulin and IGF-1 entrain circadian rhythms throughout the body by induction of PERIOD clock proteins.

## Introduction

Circadian rhythms, endogenous ∼24 h oscillations, are intrinsic to the biology of most multicellular organisms (Dunlap, 1999, Roenneberg and Merrow, 2005), controlling the temporal organization of many physiological and cellular functions (Green et al., 2008). Circadian timekeeping has a cell-autonomous basis (Balsalobre et al., 2000a, Welsh et al., 2005) with a machinery that comprises several interlinked transcriptional, translational, and post-translational feedback mechanisms. This drives the cycling auto-repression of “clock gene” transcription factors PER1/2/3 and CRY1/2 and rhythmic regulation of myriad clock-controlled genes (Takahashi, 2017).

In mammals, time of feeding and light exposure are the two main stimuli that adjust circadian rhythms to synchronize them with environmental cycles of day and night, a property known as entrainment. Environmental circadian disruption, as occurs acutely during jet lag and chronically in shift work, arises when external timing cues conflict with our internal biological oscillations. For example, human physiology is adapted to receive light and food coincidently in daytime. Nighttime feeding thus elicits conflicting cues, associated with reduced amplitude of physiological and clock gene rhythms (Archer et al., 2014, Dijk et al., 2012), and adverse metabolic and cardiovascular consequences (Salgado-Delgado et al., 2013, Scheer et al., 2009). Indeed, circadian disruption is a risk factor for several aging-linked diseases including type 2 diabetes, metabolic syndrome, and certain cancers (Reddy and O’Neill, 2010). Understanding how the body’s cellular clocks respond and adapt to changes in feeding time will inform strategies for maintaining individual fitness, supporting healthy aging, and public health policy.

How mammalian circadian rhythms sense and entrain to light is well-studied: photic cues are detected and processed by photoreceptive cells in the retina and relayed to the hypothalamic suprachiasmatic nucleus (SCN) (Berson et al., 2002, Chen et al., 2011). The SCN integrates these timing cues into its molecular and electrophysiological oscillation, then signals to cells throughout the brain and body *via* direct and indirect mechanisms, with adrenal glucocorticoids being a principal hormone communicating SCN timing with the rest of the body (Buijs et al., 1999, Welsh et al., 2010).

As with light:dark (LD) cycles, daily feeding cycles are sufficient to entrain the phase of every cell in the body, except in the SCN (Damiola et al., 2000), yet the underlying mechanism of food entrainment is not understood (Pendergast and Yamazaki, 2018). Entrainment by feeding does not require SCN input (Marchant and Mistlberger, 1997) and persists without most major components of the known molecular clockwork (Pendergast et al., 2017, Storch and Weitz, 2009). For decades, the prevailing hypothesis has centered on a “food entrainable oscillator”: a nutrient-sensing locus equivalent to, but distinct from, the SCN; although the existence and location of this locus is controversial (Mistlberger, 2009).

Given the cell-autonomous nature of biological timekeeping, we considered whether entrainment by feeding might instead be anatomically distributed. This would require both a systemic feeding signal with ubiquitously expressed receptors and for receptor activation to elicit changes in clock protein levels across diverse cell types. Such a global signal would be sufficient to communicate time-of-feeding to entrain circadian clocks in every cell individually, without a dominant coordinating locus. Here, we identify insulin and related insulin-like growth factor 1 (IGF-1) as such a signal, delineate a mechanism for its action on circadian rhythms *in vitro* and *in vivo*, and test its functional consequences in cellular and mouse models of circadian disruption.

## Results

### Insulin Determines Circadian Phase, Period, and Amplitude *In Vitro*

**Figure 1:**
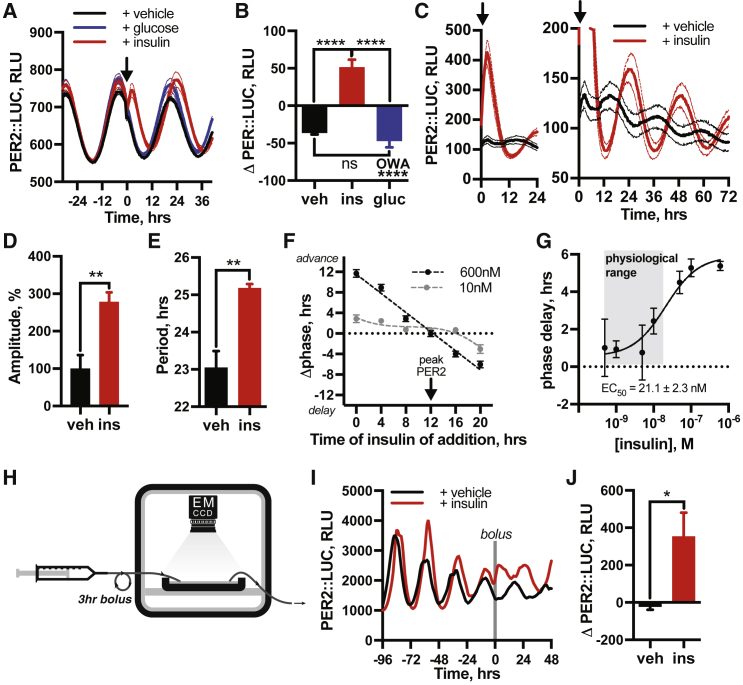
Insulin Determines Circadian Phase, Period, and Amplitude *In Vitro* (A and B) Acute addition of 600 nM insulin, but not 5.5 mM glucose, acutely increases PER2::LUC bioluminescence (ΔPER2::LUC), measured in relative luminescence units (RLU), (n = 4, one-way ANOVA, OWA, Tukey’s multiple comparisons test [MCT]). (C–F) Insulin acutely induces PER2::LUC (C), and changes the (D) amplitude, (E) period (n ≥ 4, t test), and (F) phase of PER2 expression. Phase-response curve (PRC) and preferred fit (extra sum-of-squares F test) for fibroblasts treated with 600 nM (n = 4, p <0.0001, horizontal vs. straight line fit, type 0 PRC) or 10 nM insulin (n = 6, p = 0.025, straight line vs. cubic fit, type 1 PRC). (G) Dose-response curve showing phase shift in PER2::LUC rhythm versus final insulin concentration applied 5 h after PER2 peak (n = 4). (H and I) Perfusion culture (H) with 3 h insulin bolus (I) induces PER2::LUC in fibroblasts (n = 3, representative trace shown); only here was insulin removed after addition. (J) ΔPER2::LUC by insulin under perfusion (n = 3, t test). Mean ± SEM shown for all panels except (I).

Restricted feeding cycles are sufficient to determine timing of locomotor activity and clock gene activity in peripheral tissues (Abe et al., 1989, Damiola et al., 2000). As potential timing cues, the postprandial rise in circulating glucose and insulin are obvious candidates, both being competent to affect clock gene transcription (Balsalobre et al., 2000a, Hirota et al., 2002). To determine whether insulin or glucose could affect clock protein expression, insulin or glucose was applied to PER2::LUC fibroblasts maintained in insulin-free low glucose (5.5 mM) media (Figures 1A and 1B). PER2::LUC is a valuable reporter of circadian function, as it reports translation of the endogenous clock protein PER2 (Yoo et al., 2004). While glucose had no significant effect, insulin produced a significant and rapid induction of PER2::LUC expression.

Subsequent investigation showed acute PER2::LUC induction by insulin (Figure 1C, left) was followed by significant increase in the subsequent amplitude of oscillation (Figure 1D) and also affected its period (Figure 1E). Most strikingly, insulin shifted the phase of PER2::LUC rhythms dose-dependently, with high concentrations resetting the cells to the peak of PER2 (type 0 resetting) and lower concentrations eliciting phase shifts whose direction and magnitude varied by time of insulin addition (type 1 resetting), with greatest shifts occurring at the PER2 nadir (Figure 1F). Importantly, insulin concentrations as low as 1 nM were sufficient to shift the phase of cellular rhythms (Figure 1G).

The kinetics of insulin exposure in cell culture, when it remains present in media, deviate from those *in vivo*, where insulin remains elevated for only 2–3 h after feeding. To better emulate a natural post-prandial surge, a 3 h insulin bolus was applied to cells under continuous perfusion (Figure 1H) (Crosby et al., 2017). This insulin pulse induced PER2::LUC and altered its phase comparably with static culture conditions (Figures 1I and 1J). Thus, our data suggest a secondary function for insulin signaling, where acute changes in extracellular ligand concentration affect all the essential parameters of circadian rhythms (phase, period, amplitude) in fibroblasts.

We noted the effect of insulin on cellular rhythms was consistently preceded by acute induction of PER2::LUC. Changes in PER protein abundance are necessary and sufficient to reset molecular clock phase *in vitro* and *in vivo* (Chen et al., 2009, D’Alessandro et al., 2015). Based on current mechanistic understanding of cellular circadian timekeeping (Takahashi, 2017), the increased PER protein abundance elicited by insulin adequately accounts for resetting of circadian rhythms in cultured cells.

### Insulin Induces PER2 in Primary Cells, Tissues, and Organoids *In Vitro*

**Figure 2:**
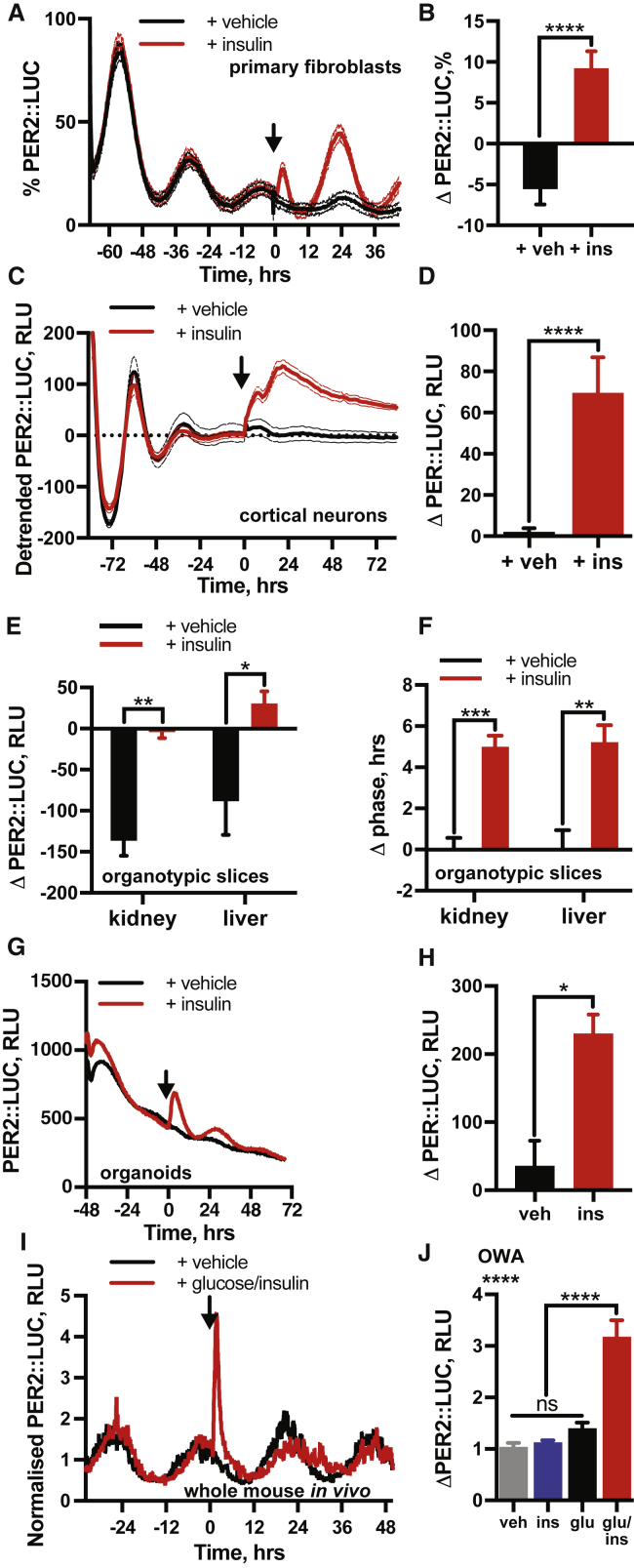
Insulin Induces PER2 in Primary Cells, Tissues, and Organoids *In Vitro* and *In Vivo* (A and B) Insulin added to primary PER2::LUC fibroblasts (A) with quantification of induction (B) (n = 4, min-max normalized, t test). (C and D) Insulin induces PER2 in dissociated PER2::LUC cortical neurons (C) with quantification of induction (D) (n = 8, one-phase exponential detrended, t test). (E and F) PER2::LUC induction (E) and phase shift by insulin (F) in mouse kidney and liver explants (n ≥ 4, t test). (G and H) Insulin acutely increases PER2::LUC in small intestinal organoids (G; n = 3, representative), with quantification of induction (H; n = 3, t test). (I and J) Combined intraperitoneal injection of insulin (2.25 U/kg) and glucose (3 g/kg) significantly increases PER2 expression after 2 h (I) (n ≥ 4, full traces in Figure S1), quantified in (J) (OWA, Dunnett’s MCT). Mean ± SEM for all panels except (G).

To assess the broader relevance of our findings, we insulin-treated primary fibroblasts (Figures 2A and 2B), dissociated cortical neurons (Figures 2C and 2D), organotypic liver and kidney slices (Figures 2E and 2F), and intestinal organoids derived from adult PER2::LUC mice (Figures 2G and 2H) (Moore et al., 2014, Sato et al., 2009). In all cases, insulin rapidly induced PER2, increased circadian amplitude, and shifted the phase of molecular rhythms. The observation that insulin acutely regulates clock protein levels and modulates circadian rhythms in many cell types *in vitro* and *ex vivo* is consistent with an additional function of this peptide messenger to communicate time-of-feeding to the molecular clock.

### Insulin Acutely Increases PER Protein Abundance *In Vivo*

**Figure S1:**
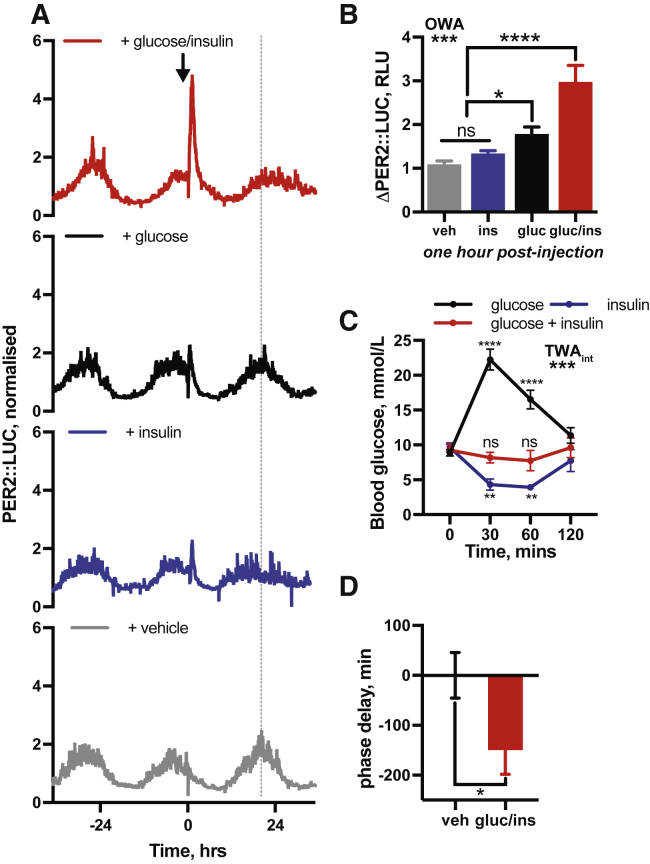
Insulin Affects Circadian Gene Expression *In Vivo,* Related to Figure 2 (A) Bioluminescent recordings of PER2::LUC mice following i.p. injection of insulin (2.25 IU/kg) or glucose (3 g/kg) or insulin and glucose in combination. Arrow indicates timing of i.p. injection. Grey line indicates timing of PER2::LUC peak in the vehicle-treated group (n ≥ 4, representative). Glucose/insulin and vehicle traces repeated from Figure 2H. (B) Quantification of the change in PER2::LUC signal at 1 h following i.p. injection (n ≥ 4, 1-way ANOVA, Tukey’s multiple comparisons test). (C) Circulating blood glucose sampled from mouse-tail following i.p. injection as in Figure S1A (n = 4, mean ± SEM, 2-way ANOVA, Dunnett’s multiple comparisons test versus t = 0 reported). (D) Quantification of difference in phase of PER2::LUC expression *in vivo* between the vehicle and insulin/glucose injected groups, from Figure S1A (Welch’s t test, one tailed). Values are relative to the vehicle-treated group.

If insulin is a bona fide systemic circadian timing cue, then acute insulin administration should induce PER2 *in vivo*, in the absence of feeding. To test this, bioluminescence was recorded from freely moving PER2::LUC mice (Saini et al., 2013). After 3 days in constant darkness, mice were dosed with vehicle, glucose (3 g/kg), insulin (2.25 IU/kg), or combined glucose and insulin, on the descending slope of PER2 bioluminescence (Figures 2I and S1A). Glucose or insulin alone transiently increased PER2::LUC activity, (Figure S1B) but the effects of neither treatment were significant after 2 h (Figure 2J). Insulin administration induces hypoglycemia, while glucose alone triggers hyperglycemia (Figure S1C). Physiologically, insulin surges to counteract an elevation in blood glucose, so combined administration of glucose and insulin better reflects changes that occur *in vivo* upon feeding (Figure S1C). Critically, combined insulin and glucose elicited rapid and sustained increases in PER2::LUC (Figure 2I) that remained elevated above controls at 2 h (Figure 2J). Furthermore, combined glucose and insulin significantly delayed timing of the subsequent PER2 peak (Figure S1D). This supports the hypothesis of a system-wide function of insulin to induce PER expression *in vivo*.

### The SCN Is Robust against Resetting by Insulin

**Figure S2:**
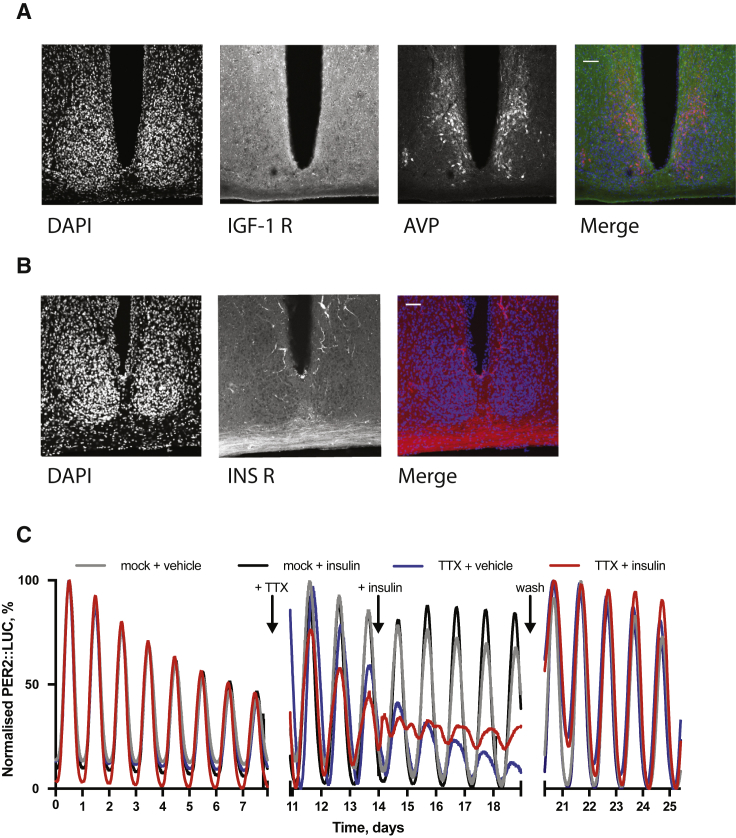
Insulin and IGF-1 Receptor Expression throughout the SCN, Related to Figure 3 (A) Immunohistochemistry for the IGF-1 receptor and (B) insulin receptor in the SCN (representative, n = 3). Scale bar represents 50 μm. (C) Addition of insulin to organotypic PER2::LUC SCN slices has no significant effect on the phase or period of oscillation. An effect on phase is observed when slices are pre-treated with tetrodotoxin (TTX) prior to insulin addition. Pre-recording 0-180 h, TTX added after 200 h, insulin added at 338 h, wash-off 490-620 h (n ≥ 3, representative, extended from Figure 3A).

The SCN is a central locus for circadian pacemaking in mammals, coordinating behavior and physiology with external 24 h light-dark cycles. Unlike the rest of the body, the SCN is insensitive to shifts in feeding schedule (Damiola et al., 2000), and this robustness against feed-fast cycles is attributed to strong interneuronal coupling (Welsh et al., 2010). Nevertheless, insulin and IGF-1 receptors are expressed throughout the SCN (Anhê et al., 2004, Bondy and Cheng, 2004) (Figures S2A and S2B). We thus expected that insulin would elicit some effect upon SCN rhythmicity.

**Figure 3:**
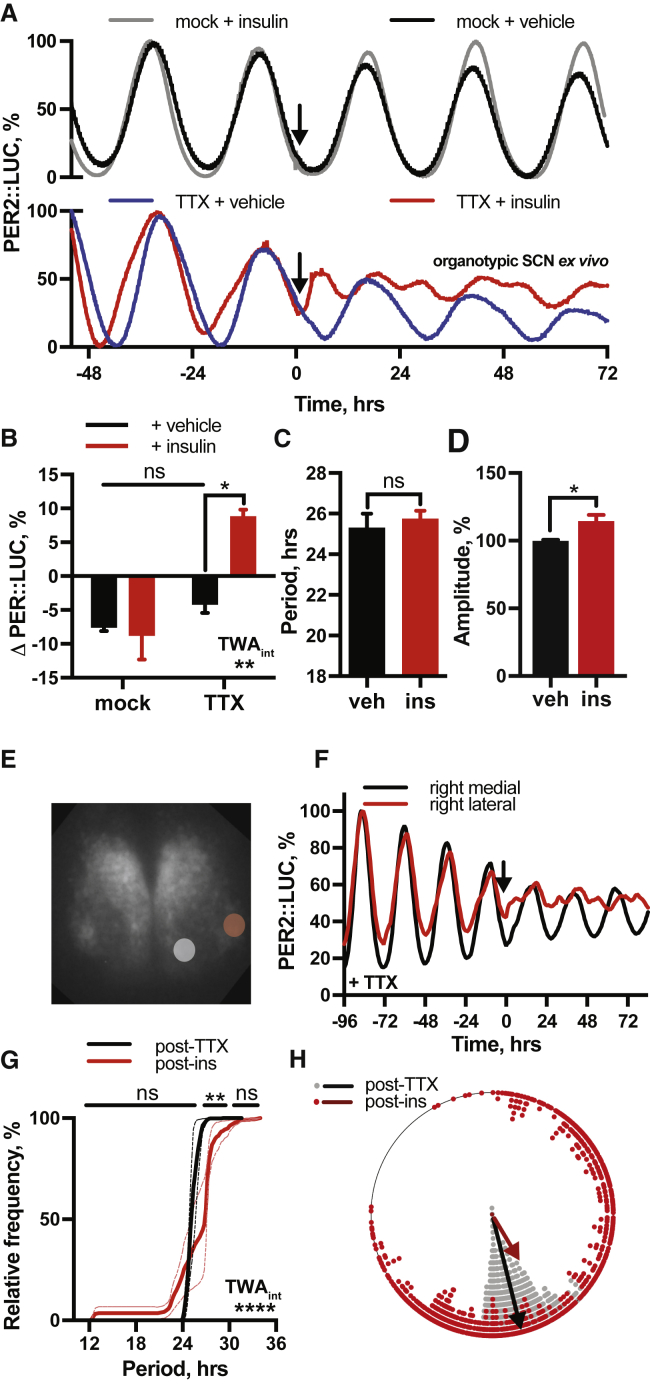
The SCN Is Robust against Resetting by Insulin (A–D) Insulin added to SCN slices (A) (top) does not (B) induce PER2::LUC (n ≥ 3, representative) or (C) alter period, although (D) amplitude is modestly increased (n = 5, t test). SCN pre-treated with tetrodotoxin (TTX) (A) (bottom) do show (B) acute PER2 induction by insulin (n ≥ 3, representative, TWA, Tukey’s MCT). See also Figure S2. (E and F) Time-lapse analysis of TTX-treated SCN (E) shows lateral SCN (red circle) is more responsive to insulin (F) (n = 3, representative). (G) Pixel analysis of this region shows cells maintain ∼24 h period following insulin with no significant increase in ∼12 h periods (n = 3, TWA, Sidak MCT). (H) Broader distribution of phases; pre-insulin = 11.05 ± 0.03 h (n = 822 pixels across 3 slices), post-insulin = 9.49 ± 0.17 h, (n = 737 pixels across 3 slices), F test variance comparison of 29.8, p < 0.0001. Mean ± SEM shown where applicable. See also Figure S3.

Organotypic SCN slices showed no acute PER2::LUC response to insulin and no change in the phase or period of PER2 rhythms (Figures 3A, top, 3B, and 3C). A small increase (14% ± 5%) in subsequent amplitude of PER2::LUC rhythms was evident (Figure 3D) suggesting that insulin has some activity in the SCN, but network coupling renders rhythms robust against the resetting seen in other cell types. To test this, SCN slices were treated with insulin in the presence, or absence, of the uncoupling Na^+^-channel blocker tetrodotoxin (TTX, 1 μM) (Yamaguchi et al., 2003). TTX pre-treatment revealed profound effects of insulin on the SCN, (Figures 3A, 3B, and S2C) with resultant waveforms now showing two distinct peaks every 24 h. Analyses of time-lapse bioluminescence microscopy recordings of TTX-treated PER2::LUC SCN revealed that the lateral region of the SCN, a locus strongly associated with pacemaker robustness (Maywood et al., 2011), was particularly sensitive to insulin (Figures 3E and 3F). Pixel analysis of this region showed that individual cells maintained their ∼24 h period of oscillation following insulin, but with a broader range of phases (Figures 3G and 3H).

### Insulin and IGF-1 Increases PER Protein Selectively

**Figure S3:**
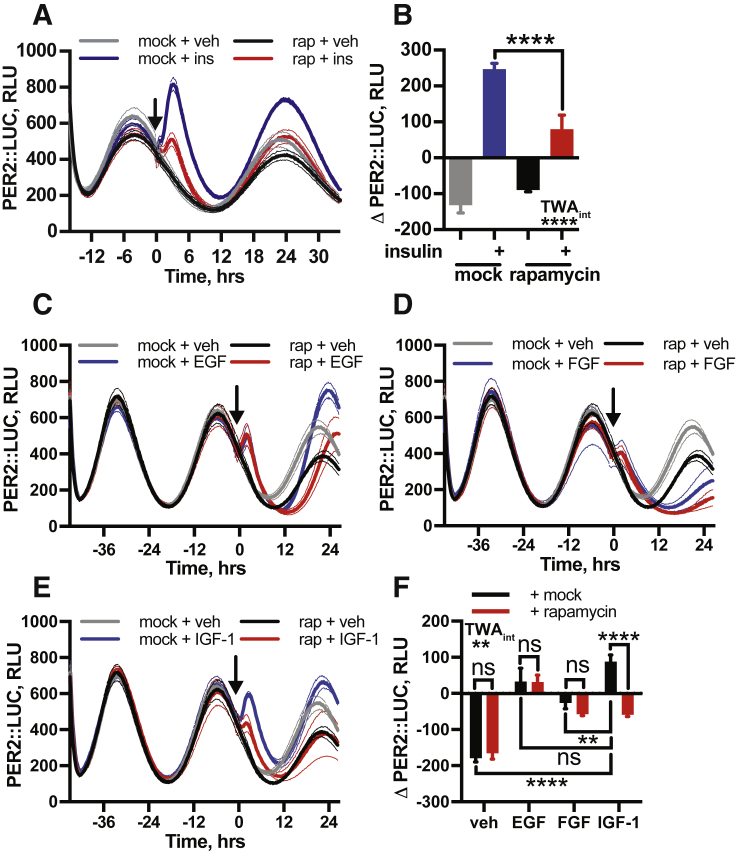
Insulin and IGF-1 Induce PER Expression through Similar Mechanisms, Related to Figures 3 and 5 (A,B) Prior treatment with mTOR inhibitor rapamycin significantly attenuates the acute PER2::LUC induction following insulin addition (n = 4, mean ± SEM, 2-way ANOVA, Tukey’s multiple comparisons test). (C) EGF and (D) FGF elicit a modest but significant induction of PER2 that is not mTOR dependent. (E) IGF-1 phenocopies mTOR-dependent induction of PER2 by insulin, demonstrated by the attenuation of this response by rapamycin. (F) Quantification of the effect of these treatments upon acute PER2 expression (n ≥ 3, mean ± SEM, 2-way ANOVA, Tukey’s multiple comparisons test). All experiments were performed in PER2::LUC fibroblasts.

We next investigated the selectivity of clock gene induction by insulin by comparing induction of PER2 by insulin with other growth factors (Figure S3). While several growth factors significantly increased PER2 levels, only insulin-like growth factor 1 (IGF-1) produced a mammalian target of rapamycin (mTOR)-dependent PER2 induction comparable to that observed with insulin (see rapamycin treatment discussion below). In the context of acute responses to feeding, insulin and free IGF-1 are highly relevant humoral signals, increasing in direct response to dietary carbohydrate and protein or fat intake, respectively (Livingstone, 2013), with functional convergence between insulin and IGF-1 signaling well-documented (Slaaby et al., 2006).

**Figure S4:**
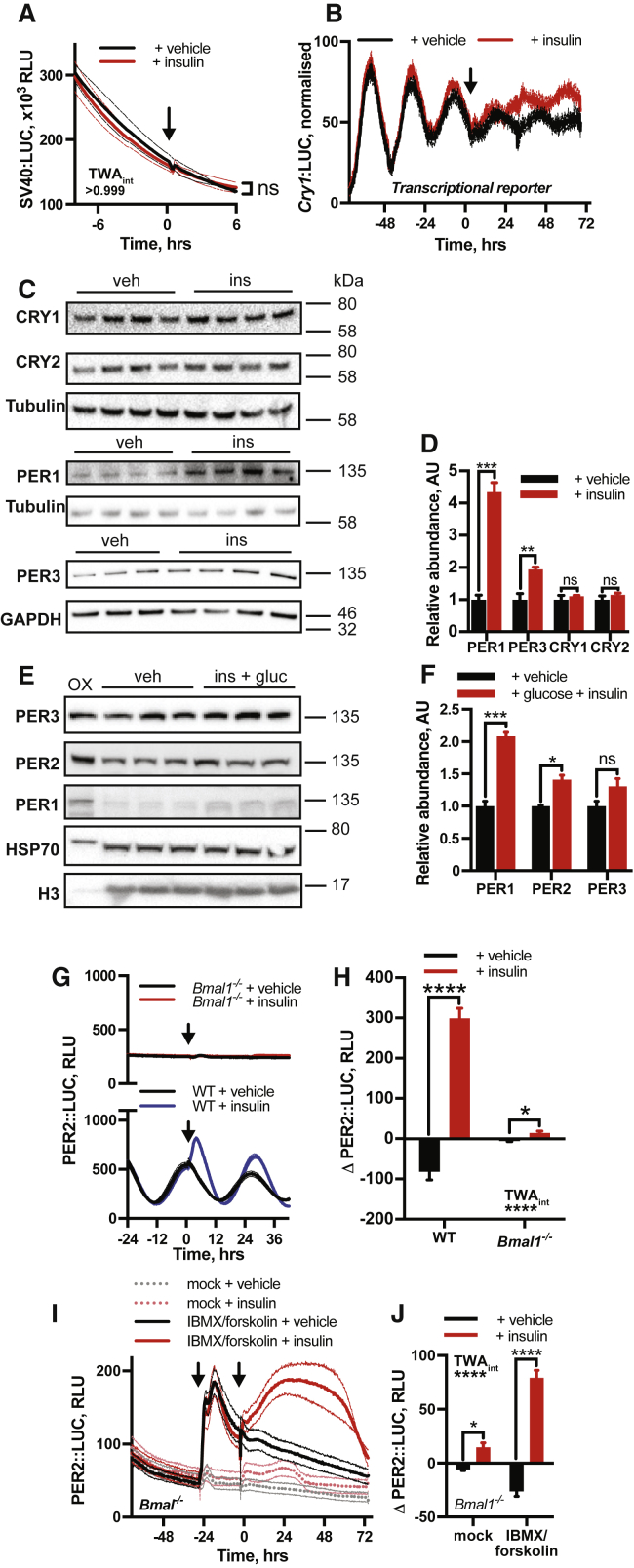
Insulin Selectively Increases PER Expression, Related to Figure 4 (A) No significant increase in luciferase expression is observed following insulin application to fibroblasts expressing luciferase constitutively under the control of the SV40 promoter (n = 3, mean ± SEM, 2-way ANOVA, Sidak’s multiple comparisons test). (B) Addition of insulin to cells expressing *Cry1*:LUC produces a phase shift but no acute induction (n = 4, mean ± SEM). (C) Western blotting on whole cell lysate from PER2::LUC fibroblasts shows a significant increase in the abundance of PER1 and PER3 following insulin treatment but no significant increase in the abundance of CRY1 or CRY2 (n ≥ 3). All samples were harvested 3 h after insulin addition. (D) Quantification of western blotting, normalized against relevant loading control (n ≥ 3, mean ± SEM, Welch’s t test). E, F Western blotting on mouse livers harvested 1 h following IP injection with glucose (3 g/kg) and insulin (2.25 IU/kg) shows a significant increase in both PER1 and PER2 abundance (n = 3, mean ± SEM, Welch’s t test), samples were normalized to histone H3 levels. Positive control (OX) in left-hand lane is extract from HEK cells transiently transfected with PER expression constructs. G,H Insulin addition to *Bmal1*^*−/−*^ PER2::LUC fibroblasts elicits a modest but significant PER2::LUC induction (n ≥ 3, mean ± SEM, 2-way ANOVA, Tukey’s multiple comparisons test). WT traces repeated from Figure 4A. I,J Combined addition of phosphodiesterase inhibitor (IBMX) and adenylyl cyclase activator (forskolin) to *Bmal1*^*−/−*^ PER2::LUC fibroblasts increases basal PER2::LUC transcription (first arrow), allowing the effect of acute insulin treatment (second arrow) to be readily observed (n = 4, mean ± SEM, 2-way ANOVA, Sidak’s multiple comparisons test).

Insulin signaling affects many aspects of cellular gene expression (Siddle, 2011), but insulin elicited no acute change in bioluminescence from fibroblasts expressing luciferase constitutively (Figure S4A) or under the control of the *Cry1* promoter (*Cry1*:LUC) (Maywood et al., 2013), CRY1 being a repressive partner to PER2 (Figure S4B) (Takahashi, 2017). This indicates that PER2::LUC induction by insulin does not reflect an increase in luciferase activity, global gene expression, or E-box-mediated clock gene transcription. Analysis by western blot showed that levels of CRY were unaffected after insulin, whereas abundance of PER2 paralogs (PER1, PER3) increased significantly (Figures S4C and S4D), indicating selective induction of PER protein expression. This is consistent with current understanding that PER abundance normally limits the rate of nascent PER/CRY complex formation (Aryal et al., 2017, Ye et al., 2014). The relative abundance of PER1 and PER2 also increased *in vivo* following intraperitoneal (i.p.) injection of insulin (Figures S4E and S4F).

**Figure 4:**
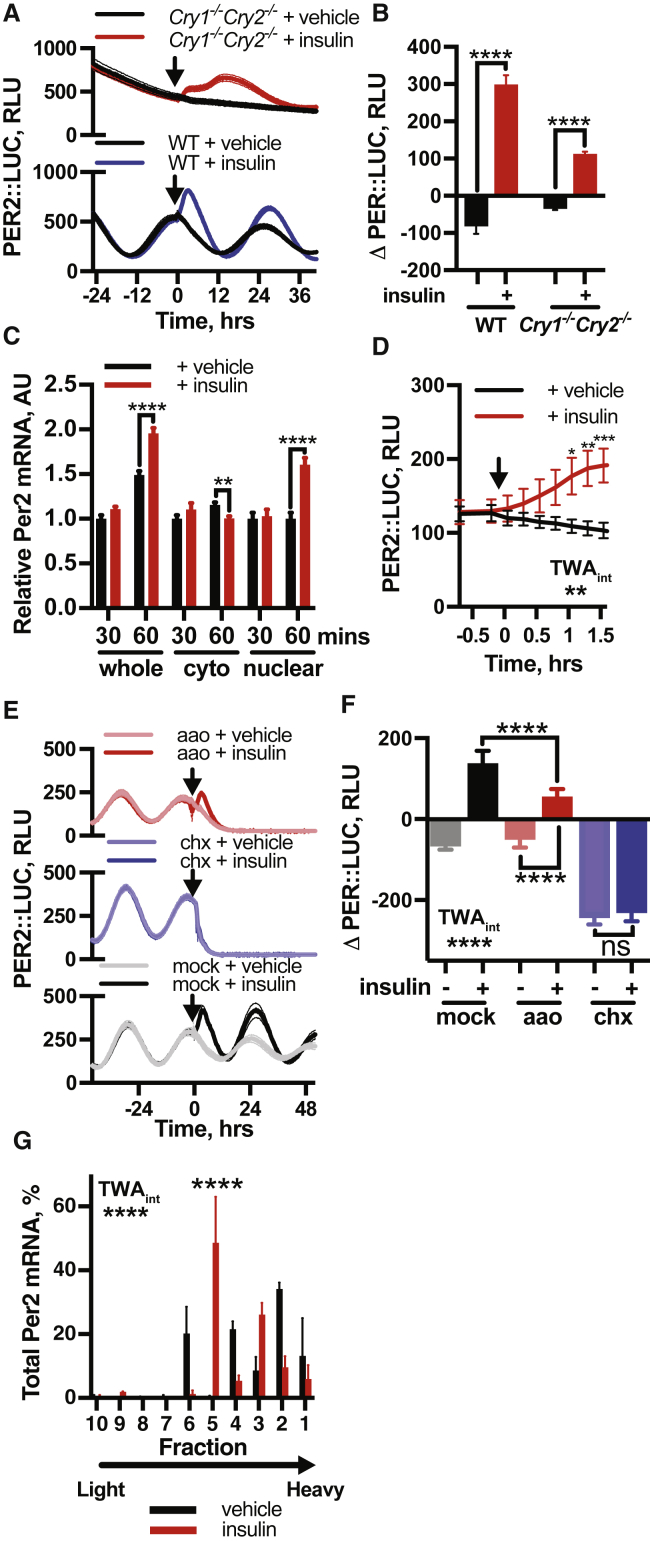
Acute Induction of PER by Insulin Is Initially Post-transcriptional (A and B) Insulin induces PER2 in *Cry1*^−/−^*Cry2*^−/−^ PER2::LUC fibroblasts (A), with quantification of induction (B) (n ≥ 3, t test). (C) qPCR on subcellular fractions of PER2::LUC fibroblasts post-insulin show no significant increase in *Per2* mRNA at 30 mins and no increase in the cytoplasmic fraction at 60 min (n = 5, multiple t tests). (D) Increased PER2::LUC in fibroblasts within 1 h of insulin treatment (n = 4, TWA, Sidak MCT). (E and F) Inhibition of transcription (aao) attenuates PER2::LUC induction, whereas translational inhibition (chx) abolishes PER2::LUC induction by insulin (E), with quantification of induction (F) (n ≥ 3, TWA, Tukey’s MCT). (G) Polyribosome fractionation analyzed by qPCR shows altered distribution of *Per2* mRNA 60 min after insulin addition (n = 3, TWA, MCT). See also Figure S5F. Mean ± SEM shown throughout. See also Figure S4.

In mice, circadian entrainment to feeding cycles persists without most components of the core circadian transcriptional machinery (Storch and Weitz, 2009), so we tested for PER2 induction by insulin in cells lacking these critical factors. In *Cry1*^−/−^/*Cry2*^−/−^ and *Bmal1*^−/−^ fibroblasts, we found insulin also elicited PER2 induction (Figures 4A, 4B, S4G, and S4H), indicating that the canonical transcriptional clock mechanism is not required for this activity. We speculate that in *Cry1*/*2*- and *Bmal1*-deficient mice, consistent with findings by Mauvoisin et al. (2014), daily feeding-driven rhythms of insulin-stimulated PER synthesis are sufficient to drive downstream daily gene expression patterns, functioning as an interval timer, without requiring cell-autonomous gene expression rhythms.

### Acute Induction of PER2 by Insulin Is Initially Post-transcriptional

For the quantity of a cellular protein to increase, one of three processes must occur: an increase in transcription and subsequent translation, increased translation of existing mRNA, or decreased protein degradation. Increases in PER abundance during the circadian cycle are largely attributed to increasing *Per2* mRNA levels (Takahashi, 2017). In contrast, qPCR analysis of insulin-treated cells showed no significant increase in cytoplasmic *Per2* mRNA within 1 h of insulin application (Figure 4C), despite PER2::LUC increasing during this time (Figure 4D). We therefore investigated the mechanism of PER induction using pharmacological inhibitors of transcription (α-amanatin oleate [aao]), translation (cycloheximide [CHX]), and proteasomal degradation (MG132).

**Figure S5:**
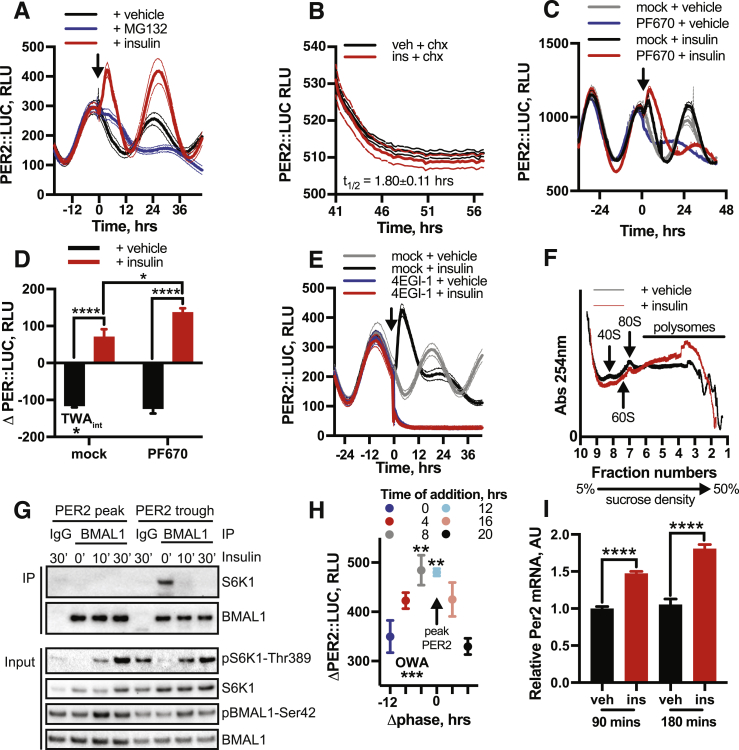
Initial Induction of PER Occurs through Increased Translation of Existing mRNA, Related to Figure 4 (A) Inhibition of PER2 degradation with proteasomal inhibitor MG132 (n = 4, mean ± SEM) does not replicate the acute induction of PER2::LUC following insulin treatment (n = 4, mean ± SEM). (B) Pre-treatment with insulin does not influence PER2 degradation rate following cycloheximide, with both decay curves sharing the same half-life (n = 4, mean ± SEM, extra-sum-of-squares F test, p = 0.99), indicating that insulin does not increase PER2 levels by decreasing its rate of degradation. C, D Casein kinase 1 inhibitor PF-670462 also fails to replicate the PER2 induction following insulin (n = 4, mean ± SEM, 2-way ANOVA, Tukey’s multiple comparisons test). Consistent with insulin triggering an increase in PER2 synthesis however, we note that PF-670462 further potentiates the PER2::LUC induction following insulin treatment. (E) Inhibition of cap-dependent translation with 4EGI-1 abolishes the PER2 induction by insulin (n = 4, mean ± SEM). (F) Representative polysome profiles of absorbance at 254 nm at 60 min following treatment with vehicle or insulin (n = 3, representative). 40S, 60S and 80S peaks, and polysomes, are indicated. (G) Co-immunoprecipitation of S6K and BMAL1 at 10 and 30 min following insulin treatment shows a decrease in association of these proteins in response to insulin treatment, suggesting that BMAL1 association with translational machinery does not contribute to the acute increase in PER2 translation that follows insulin treatment. (H) Quantification of the magnitude of the PER2::LUC induction against shift in phase shows the largest induction when insulin is applied at the time when *Per2* mRNA is most abundant (at 4 h before and around peak PER2::LUC), with significantly smaller inductions at the trough of PER expression, when *Per2* mRNA is less abundant (n = 4, mean ± SEM, 1-way ANOVA, Dunnett’s multiple comparisons test, p-value versus the first group is shown). (I) An increase in whole-cell *Per2* mRNA levels is observed at 90 and 180 min following insulin addition (n = 4, mean ± SEM, Welch’s t test). Taken together, these data suggest that, although increased PER2 transcription may contribute to some of the insulin-induced increase in PER2, increased PER translation from existing mRNA is primarily responsible for the acute increase in PER levels following insulin treatment, while the stability of PER is unaffected. All experiments were performed in PER2::LUC fibroblasts.

Rapid induction of PER2::LUC by insulin was maintained under blockade of *de novo* transcription (Figures 4E and 4F), showing that nascent transcription is not required for increased PER synthesis. Inhibition of proteasomal degradation did not increase PER2 levels similarly to insulin (Figure S5A) nor did insulin affect PER2 stability (Figure S5B). Furthermore, the effect of insulin was not replicated by inhibition of casein kinase 1(CK1)-directed PER protein degradation (PF670462) (Meng et al., 2010) (Figures S5C and S5D), rather this enhanced PER2 induction by insulin. Finally, administration of CHX completely abolished PER2::LUC induction by insulin (Figures 4E and 4F), as did inhibition of CAP-dependent translational initiation (Figure S5E).

We thus conclude that initial increases in PER protein following insulin are primarily due to increased translation of existing *Per* mRNA and not increased mRNA production or decreased protein degradation. This contrasts with other factors that are known to increase PER protein production by transcriptional activation (Balsalobre et al., 2000a, Balsalobre et al., 2000b, Yan et al., 2008). Our interpretation is supported by three further observations. First, polysome profiling showed significant shift in *Per2* mRNA distribution 1 h after insulin treatment (Figures 4G and S5F), indicating differential PER2 translation. Second, insulin engendered a modest increase in PER2 levels in cells lacking *Bmal1*, a transcription factor essential for E-box-mediated *Per2* transcription (Figures S4G and S4H). However, pre-treating *Bmal1*^−/−^ cells with forskolin and IBMX to increase *Per2* transcription via Ca^2+^ and cAMP promoter response elements produced greatly increased PER2 levels after insulin (Figures S4I and S4J). This suggests that availability of pre-existing *Per2* mRNA determines the magnitude of subsequent PER2 induction by insulin. BMAL1 can also potentiate protein synthesis through association with S6K1 (Lipton et al., 2015); although we found insulin addition to wild-type cells actually reduced this association (Figure S5G). Finally, we noted strong phase-dependence of the magnitude of PER2 induction by insulin in wild-type cells (Figure S5H), consistent with increased translational efficiency from a transcript whose abundance varies over the circadian cycle.

While transcriptional activation cannot account for the initial surge of PER2 production, *Per2* mRNA was elevated after 90 min of insulin treatment (Figure S5I), and PER2 induction was attenuated by transcriptional inhibition (Figure 4E). The kinetic discrepancy between rapidly increased PER2::LUC and slower increases in *Per2* mRNA therefore suggests a bipartite response to insulin, with initial increases in PER protein expression representing increased translation of existing *Per* transcript, followed by a slower increase in mRNA levels.

### Coincidence Detection Facilitates Selective PER Induction by Insulin

**Figure S6:**
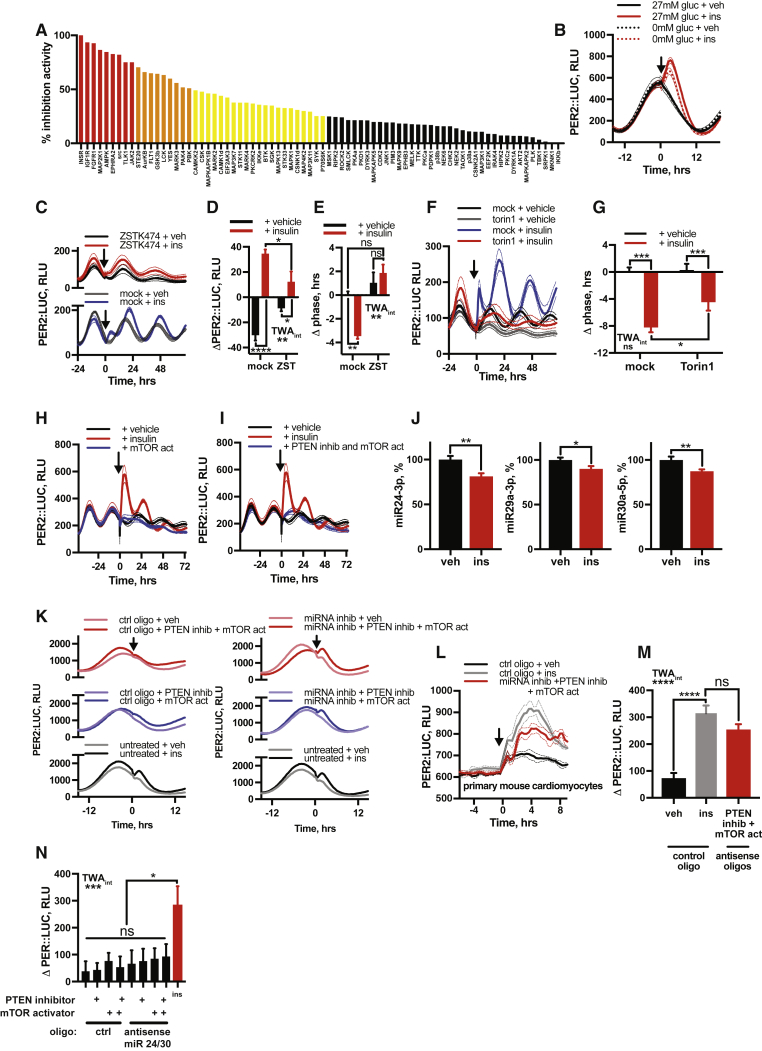
Selective PER Induction Requires Coincident Intracellular Signals, Related to Figure 5 (A) Kinase inhibition profile for BMS-754807 across a panel of protein kinases at 1 μM test concentration. Results are expressed as average percentage inhibition. Red bars indicate greater than 75% inhibition, orange greater than 50% and yellow greater than 25%. (B) Addition of insulin (600 nM) in the absence of extracellular glucose elicits a clear induction of PER2::LUC, which is potentiated by the presence of glucose extracellularly (n = 4, mean ± SEM). See Figure 5D for quantification. (C,D) Inhibition of PI3K (ZSTK474) abolishes both the acute induction of PER2 following insulin and (E) the subsequent shift in circadian phase (n = 4, mean ± SEM, 2-way ANOVA, Tukey’s multiple comparisons test). Please note that the effect of LY294002 on phase cannot be analyzed, since it abolishes PER2::LUC expression within 24 h of application. (F,G) Inhibition of mTOR with torin1 significantly attenuates the phase shift evoked by insulin (n = 4, mean ± SEM, 2-way ANOVA, Tukey’s multiple comparisons test). (H) mTOR activator MHY1485 does not induce PER2 expression comparably to insulin (n = 4, mean ± SEM), and nor does (I) simultaneous application of MHY1485 and PTEN inhibitor VO-OHpic (n = 4, mean ± SEM). (J) qPCR analysis shows levels of miR24-3p, miRNA29a-1 and miR30a-5p are all significantly reduced in PER2::LUC fibroblasts after 60 min of insulin treatment (n = 4, mean ± SEM, Welch’s t test). (K) Simultaneous inhibition of miRs 24-3p, 29a-1 and 30a-5p, pharmacological inhibition of PTEN and activation of mTOR in PER2::LUC fibroblasts recapitulates the PER2 induction by insulin, an effect not seen with any of these treatments alone or in dual combination (n = 4, representative). Extended from Figure 5I. (L,M) miRNA inhibition combined with PTEN inhibition and mTOR activation recapitulates the PER2 induction by insulin in PER2::LUC cardiomyocytes (n ≥ 4, mean ± SEM, 2-way ANOVA, Tukey’s multiple comparisons test). (N) In fibroblasts, silencing of only miR 24-3p and mIR 30a-5p, but not mIR 29a-1, combined with inhibition of PTEN and activation of mTOR does not induce PER2::LUC comparably with insulin (n = 4, mean ± SEM, 1-way ANOVA, Tukey’s multiple comparisons test).

**Figure 5:**
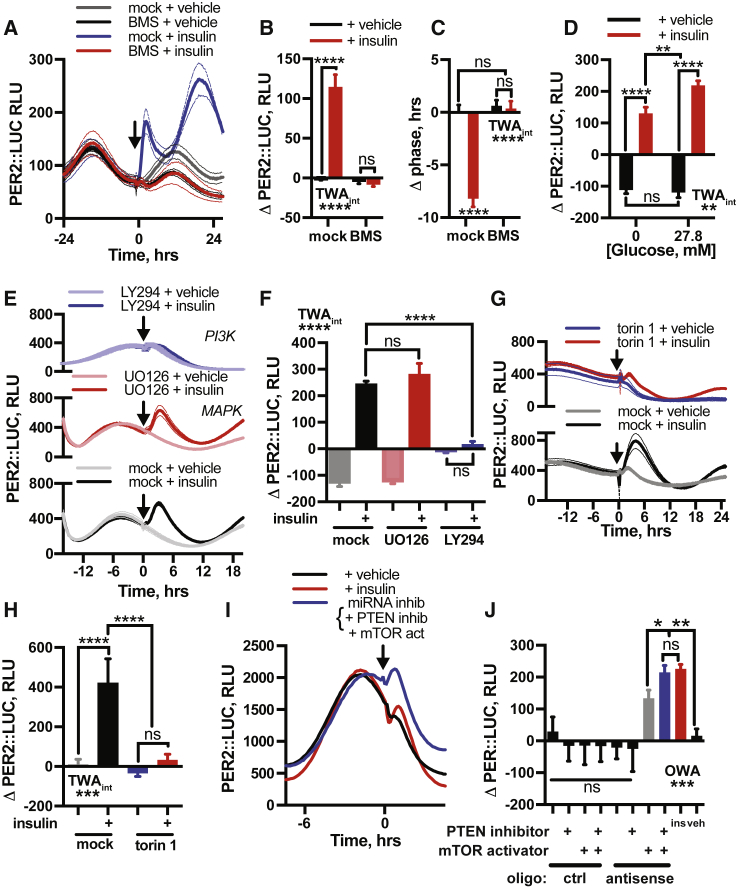
Coincidence Detection Facilitates Selective PER Induction by Insulin (A–C) IR and IGF-1R antagonist (A) BMS-754807 abolishes PER2::LUC induction (B) and phase shift (C) by insulin in fibroblasts (n = 4, TWA, Tukey’s MCT). (D) Extracellular glucose potentiates, but is not required for, PER2::LUC induction by insulin (n = 4, TWA, Tukey’s MCT). See also Figure S6B. (E and F) Application of MAPK pathway inhibitor U0126 does not affect PER2 induction by insulin, while inhibition of PI3K (LY294002) abolishes it (E), with quantification of induction (F) (n ≥ 3, TWA, Tukey’s MCT). (G and H) Inhibition of mTOR (torin 1) attenuates PER2 induction by insulin (G), with quantification of induction (H) (n = 4, TWA, Tukey’s MCT). (I and J) Simultaneous inhibition of miR24-3p, miR29a-1, and miR30a-5p with PTEN inhibition and mTOR activation recapitulates PER2::LUC induction by insulin in fibroblasts (I) (n = 4, representative, see also Figure S6K), with quantification (J) (OWA, Tukey’s MCT). Mean ± SEM for all panels except (I). See also Figure S5.

Having found that insulin stimulates PER synthesis *in vivo*, in tissues *ex vivo*, and in cultured cells *in vitro*, we considered the basis of signaling selectivity for increased PER translation. Given the functional convergence of insulin and IGF-1 upon insulin and IGF-1 receptors, we first tested whether PER induction by insulin is dependent upon insulin and IGF-1 receptor kinase activity by pre-incubating fibroblasts with BMS-754807, an ATP-competitive antagonist of IR and IGF-1R (Figure S6A) (Carboni et al., 2009). We found IR and IGF-1R inhibition abolished PER2 induction by insulin and the subsequent phase shift (Figures 5A–5C), consistent with IR and IGF-1R activation stimulating PER synthesis.

Cellular circadian rhythms are sensitive to glucose availability and rates of primary metabolism (Bass, 2012, Hirota et al., 2002, Putker et al., 2018). Glucose uptake is one consequence of insulin signaling (Leney and Tavaré, 2009), and maximal sustained induction of PER2 required co-treatment with glucose *in vivo* (Figures 2I and 2J). PER2::LUC induction by insulin was observed in cells maintained in glucose-free media, however (Figures 5D and S6B), suggesting that other effects of IR and IGF-1R activation, besides increased glucose uptake, are critical to translational regulation of PER. Extracellular glucose did modestly potentiate PER2 induction, suggesting glucose metabolism can modulate, but is not essential for, insulin’s action on circadian rhythms.

Ligand binding at IR or IGF-1R activates signal transduction by both mitogen-activated protein kinase (MAPK) and phosphatidylinositol-3-kinase (PI3K) families, each with many downstream targets (Siddle, 2011). We inhibited each pathway pharmacologically to determine their contribution to PER induction and subsequent phase shifts. While MAPK inhibition (UO126) had no significant effect, PI3K inhibition (LY294002, ZSTK474) profoundly attenuated PER2 induction and phase shifts by insulin, suggesting PI3K as the primary effector from the IR and IGF-1R receptors to the cellular clock (Figures 5E, 5F, and S6C–S6E).

IR and IGF-1R activation increases the phosphatidylinositol(3,4,5)-triphosphate (PIP_3_) pool, both directly by activating PI3K, and by downregulating phosphatase and tensin homolog (PTEN) activity (Taniguchi et al., 2006). This leads to activation of mTOR complex 1 (mTORC1), a critical regulator of protein synthesis (Dibble and Manning, 2013, Shimobayashi and Hall, 2014). Pharmacological inhibition of mTOR via torin 1 or rapamycin strongly attenuated PER2 induction by insulin or IGF-1 but not other growth factors (Figures 5G, 5H, and S3), consistent with their functional convergence. mTOR inhibition also diminished insulin-evoked phase shifts (Figures S6F and S6G), although not as completely as IR and IGF-1R inhibition. However, acute activation of mTOR alone was insufficient to induce PER2 (Figure S6H), even in combination with PTEN inhibition (Figure S6I). This suggests that, while PI3K and mTOR activation are necessary for PER induction *in vitro*, they are not sufficient.

**Figure 6:**
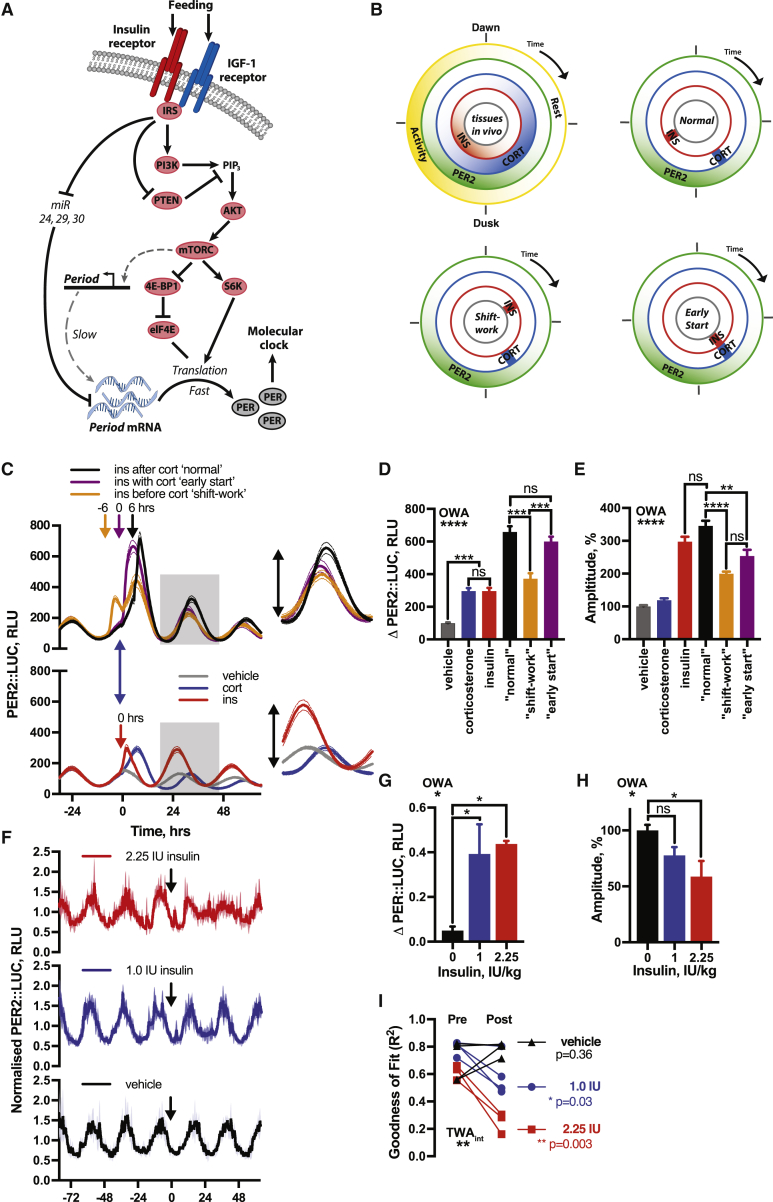
Conflicting Temporal Cues Impair Circadian Fidelity *In Vitro* and *In Vivo* (A) Proposed mechanism by which insulin induces PER expression. (B) Temporal relationship between glucocorticoid (corticosterone) and insulin profiles *in vivo* compared to *in vitro* assays. (C–E) Insulin added 6 h before (orange), 6 h after (black), or at the same time as (purple) corticosterone (C) significantly affects (D) PER2 induction and (E) subsequent PER2::LUC amplitude (red: insulin alone, blue: cort alone). Grey boxes and inset in (C) show peak used for quantification (n ≥ 3, OWA, Tukey’s MCT, see also Figure S7). (F) Bioluminescence from PER2::LUC mice i.p. injected with insulin:glucose (1.0 IU/kg:1.3 g/kg or 2.25 IU/kg:3 g/kg) during the inactive phase (n ≥ 3). (G and H) Both doses increased PER2::LUC (G) (OWA, Dunnett’s MCT) and higher doses significantly decreased the amplitude of next circadian cycle (H). (OWA, Tukey’s MCT). (I) Both doses reduced the robustness of PER2::LUC rhythms after injection, assessed by cosinor goodness of fit, 36 h prior to and following treatment (R^2^ for replicates shown, TWA, Sidak MCT). Mean ± SEM shown.

We thus considered what other consequence of insulin signaling might act with mTORC1 to increase PER translation. Translation of *Per1* and *Per2* mRNA is regulated by three microRNAs (miRNAs), namely miRNAs 24-3p, 29a-3p, and 30a-5p (Chen et al., 2013), with miR29a-3p also predicted to regulate *Per3* (Wong and Wang, 2015). It is also known that many miRNAs, including those regulating PER, are rapidly downregulated by insulin (Granjon et al., 2009, Marzi et al., 2016). These *Per*-cognate miRNAs were also downregulated in fibroblasts following insulin (Figure S6J). Moreover, selective inhibition of these miRNAs, combined with mTOR activation and PTEN inhibition, elicited an acute induction in PER2 expression that replicated induction of PER2 by insulin in both PER2::LUC fibroblasts and cardiomyocytes (Figures 5I, 5J, and S6K–S6M). Strikingly, only with inhibition of all three miRNAs was mTOR activation and PTEN inhibition sufficient to replicate this induction (Figure S6N). These observations suggest a model where the post-prandial surge in circulating insulin and IGF-1 signals via PI3K activation and PTEN inhibition to activate mTORC1 and also reduces PER-cognate miRNA levels, stimulating increased PER protein synthesis from pre-existing cytosolic mRNA (Figure 6A). Such coincidence detection, at the level of clock protein synthesis, facilitates the faithful communication of feeding time to the cellular clockwork, preventing erroneous clock resetting when TOR complexes are activated in other contexts.

### Conflicting Temporal Cues Impair Circadian Fidelity *In Vitro* and *In Vivo*

In natural environmental cycles, SCN-stimulation of the hypothalamic-pituitary-adrenal axis increases glucocorticoid (CORT) production 4–6 h prior to the onset of increased feeding and locomotor activity, both in mice and humans (Shamsi et al., 2014) (Figure 6B). This CORT profile synchronizes cells throughout the body with the external light cycle (Schibler et al., 2015), primarily by increasing *Per* gene transcription (So et al., 2009, Yurtsever et al., 2016). Consistent with this, we found that stimulation of fibroblasts with CORT shifted the cellular clock to a phase ∼4 h before peak PER2 (Figure 6C, bottom), equivalent to late in the rest phase, when clock-regulated glucorticoid activity would be maximal *in vivo*. As shown above, acute stimulation with physiological insulin concentrations shifts cellular clock phase toward the PER2 peak (Figures 1C and 1F), equivalent to early in the active phase, when the greatest surge in insulin and IGF-1 normally occurs *in vivo* at the transition from fast to feeding (Shamsi et al., 2014).

**Figure S7:**
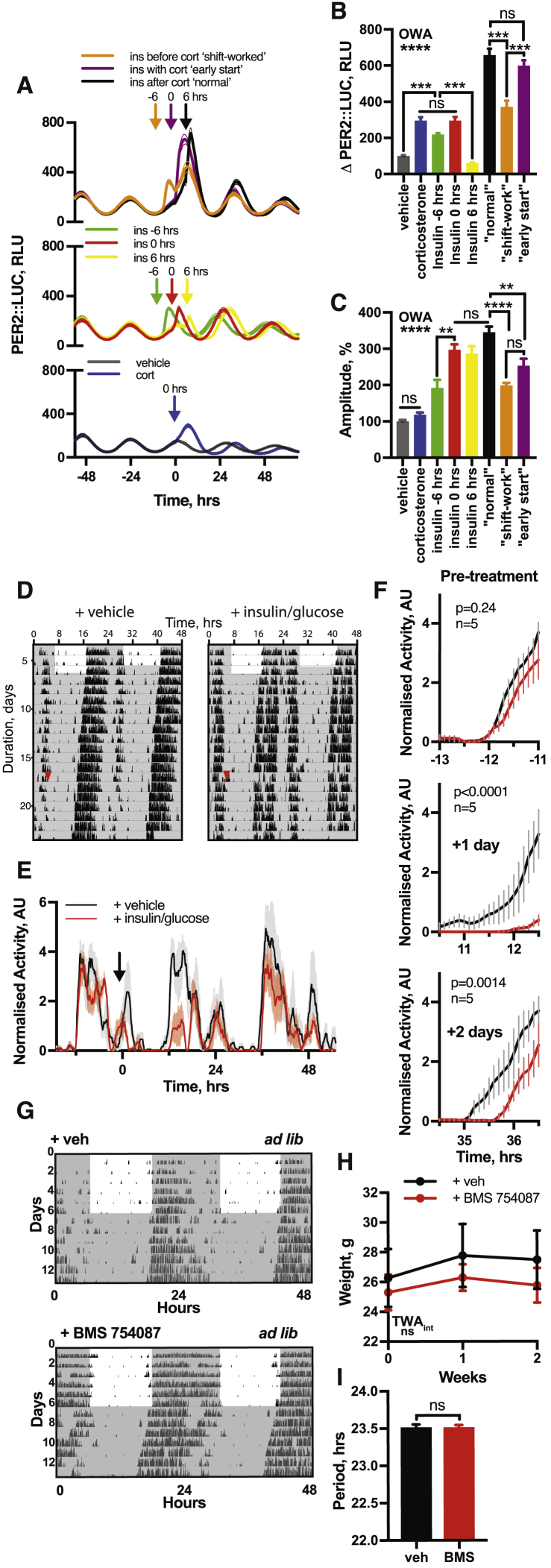
Conflicting Entrainment Cues Impare Circadian Rhythmicity, Related to Figures 6 and 7 (A) Full PER2::LUC traces from Figure 6C (n = 4, mean ± SEM). (B) Quantified PER2::LUC induction of all conditions from Figure 6C (n = 4, mean ± SEM, one-way ANOVA, Tukey’s multiple comparisons test). (C) Quantified change in amplitude of all conditions from Figure 6C (n = 4, mean ± SEM, one-way ANOVA, Tukey’s multiple comparisons test). (D) Locomotor activity of mice in constant darkness receiving combined insulin (2.25 IU/kg) and glucose (3 g/kg) or vehicle by i.p. injection. i.p. was given at the beginning of the inactive phase (n = 5, representative, red arrow indicates time of injection). Activity was measured using a beam break detection floor grid. (E) Mean average behavior of both groups before and after insulin addition (mean ± SEM). Arrow indicates time of i.p injection. (F) Expanded view showing the onset of behavior in both groups highlights that mice receiving insulin/glucose at a biologically inappropriate time exhibit both a delay and a reduction in the amplitude of the onset of activity, at both one and two days following i.p. (2-way ANOVA, p-value of interaction is reported). (G) Mice continuously fed with either IR/IGF-1R antagonist BMS-754807 or a vehicle in the drinking water (n = 6) under both LD and DD conditions. Representative actograms show wheel-running activity double-plotted along x axis. (H) No significant difference in body weight was observed between the vehicle or BMS-754807 groups (n = 6, mean ± SEM, 2-way ANOVA, Sidak’s multiple comparisons test). (I) BMS-754807 had no significant effect on circadian period of rest-activity cycles when fed *ad lib* in constant conditions (n = 6, mean ± SEM, Welch’s t test).

During shift-work, when individuals receive conflicting temporal cues (i.e., light versus food), circadian gene expression rhythms are damped (Archer et al., 2014, Dijk et al., 2012). Reduced amplitude of circadian gene expression indicates impaired biological timekeeping and is associated with diseased states (Lucassen et al., 2016, Scheer et al., 2009). If insulin signaling constitutes a bona fide primary signal of feeding time to cellular clocks throughout the body, then inversion of the 4–6 h interval between the CORT surge (increases *Per* transcription) (Le Minh et al., 2001) and the insulin and IGF-1 surge (increases PER translation) should mimic the reduction in clock gene amplitude that accompanies circadian misalignment (Shamsi et al., 2014) (Figure 6B). Accordingly, CORT application in fibroblasts followed by insulin (“normal”) elicited a greater induction of PER2::LUC and greater subsequent amplitude of oscillation than either stimulus alone, or when the sequence was inverted (mimicking “shift-work”) or when CORT and insulin were administered simultaneously (mimicking an “early start”) (Figures 6C–6E and S7A–S7C).

To verify physiological relevance *in vivo*, PER2::LUC mice in constant darkness were administered with combined insulin:glucose (1.0 IU/kg:1.3 g/kg or 2.25 IU/kg:3 g/kg) at the beginning of the rest phase, when insulin would normally be low (Figure 6F). Both doses evoked an immediate increase in PER2::LUC, as previously described (Figures 2I and 2J), but now with disorganized and lower amplitude PER2::LUC rhythms over the next 36 h (Figures 6G and 6H). The daily onset of activity of mice in constant darkness was also significantly disturbed in the 36 h following i.p. injection of insulin:glucose (2.25 IU/kg:3 g/kg) (Figures S7D–S7F) when delivered early in the rest phase. These results are consistent with a model where temporally incoherent timing cues reduce the amplitude and robustness of circadian rhythms at both cellular and organismal scales.

### IR and IGF-1R Inhibition Attenuates Entrainment of Circadian Rhythms to Feeding Time *In Vivo*

The contribution of feeding to circadian behavioral organization *in vivo* can be dissected by restricted feeding (RF), where food is available for the same few hours each day. If food is only available during an animal’s habitual inactive phase, the timing of locomotor activity shifts to coincide with feeding. This temporal reorganization of rest-activity is SCN-independent and persists after animals are returned to *ad libitum* (*ad lib*) feeding (Takasu et al., 2012), indicating endogenous timing mechanisms that respond to feeding time and organize behavior accordingly (Mistlberger, 2009).

Genetic ablation of insulin and IGF-1 signaling has pleiotropic and profound adverse effects (Kadowaki, 2000); whereas the IR and IGF-1R inhibitor BMS-754807 is orally available and well tolerated *in vivo* without major effects on glucose homeostasis, body weight, or daily rest-activity cycles (Figures S7G–S7I) (Carboni et al., 2009, Hou et al., 2011). If insulin and IGF-1 signaling communicates feeding time to cellular circadian clocks throughout the brain and body, then chronic application of BMS-754807 *via* drinking water should impair entrainment of both molecular and behavioral circadian rhythms to feed-fast cycles *in vivo*.

**Figure 7:**
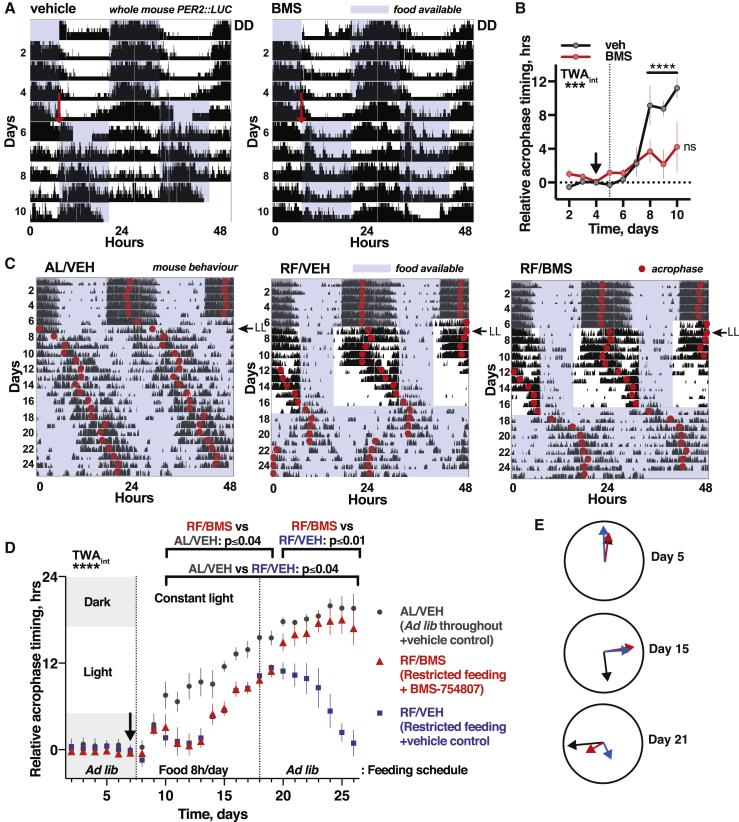
IR and IGF-1R Inhibition Attenuates Entrainment of Circadian Rhythms to Feeding Time *In Vivo* (A) Representative PER2::LUC bioluminescence for restricted fed (RF) mice, with 12 h delay in feeding time from day 5 (red arrow). BMS-754807 or vehicle provided in drinking water throughout. (B) Shift in PER2::LUC acrophase following change in feeding schedule was significantly delayed for BMS-treated group, reported relative to acrophase on days 2–4 (n = 4, TWA, Tukey’s MCT). (C) Mean wheel-running activity for mice entrained to 12 h:12 h LD cycles, then released to constant light (LL). Restricted feeding (RF) groups (n = 6) were fed 8 h/day for 9 days before return to *ad lib* feeding, with one RF group receiving BMS-754807 in drinking water from day 7. Control group freely fed throughout (left: n = 6). (D) IR and IGF-1R inhibition attenuates temporal reorganization of daily rest-activity cycles after, not during, restricted feeding under LL (n = 6, TWA, Sidak MCT). Acrophase calculated relative to acrophase on final day of LD (black arrow). (E) Mean acrophase before, during, and after RF (days 5, 15, and 21). Arrow lengths are inversely proportional to SEM. See also Figure S7.

Testing the former, PER2::LUC mice were entrained by 12 h:12 h light:dark (LD) cycles (fed *ad lib*) then released to constant darkness (day 1) with food available for 12 h each day during the habitual active phase (subjective night), with 4 out of 8 animals receiving 500 μM BMS-754807 continuously in drinking water. After 4 days, food availability was shifted by 12 h. In the control group the acrophase (peak timing) of PER2::LUC bioluminescence gradually shifted to match feeding time by around day 10, consistent with previous reports that feeding time dominates SCN cues in these conditions (Saini et al., 2013). Consistent with prediction, BMS-treatment profoundly attenuated re-organization of PER2::LUC rhythms when feeding time was inverted (Figures 7A and 7B).

We then asked whether insulin and IGF-1-dependent timing cues were sufficient to determine timing of rest-activity *in vivo*. To unmask communication of feeding time to (extra-SCN) behavioral control centers, after 1 week of standard 12 h:12 h LD entrainment, animals were transferred to constant light (LL), where SCN signaling is severely damped (Chen et al., 2008b, Ohta et al., 2005). Two experimental groups (each n = 6) underwent daily RF, with food available for 8 h/day during the rest phase (anticipated day). One group (RF/BMS) was given 500 μM BMS-754807 continuously in the drinking water from day 7, and both RF groups were returned to *ad lib* feeding 10 days later. This allowed assessment of whether IR and IGF-1R inhibition affected the re-organization of behavior elicited by daily feeding cues, compared with the control group (AL/VEH), which was maintained with constant light and food from day 7.

In the control group, activity abruptly delayed upon transition to LL and then became later each day, consistent with “Aschoff’s rule” (Chen et al., 2008b, Ohta et al., 2005). Under RF, the acrophase of activity took ∼1 week to consolidate around feeding time, with no difference between RF groups (RF/VEH or RF/BMS), and both RF groups’ acrophase occurring before controls (Figures 7C–7E). Upon return to *ad lib* feeding, however, a striking difference emerged between the RF groups: while the activity of vehicle-treated RF mice remained consolidated around the previous feeding window for ∼4 cycles before advancing, the activity phase of BMS-treated animals delayed to match that of control *ad lib* fed animals within 2 days. This is consistent with insulin and IGF-1 being the messengers of feeding time to extra-SCN brain regions, and dominant over SCN signals under LL, as IR and IGF-1R inhibition prevented stable entrainment to feeding time.

These final two experiments indicate that, in the absence of competing cues, time of feeding is sufficient to determine the temporal organization of daily rest-activity cycles and peripheral clock gene expression rhythms, and that insulin and IGF-1 signaling is necessary for circadian entrainment to feeding time in both cases.

## Discussion

We demonstrate that the hormones insulin and IGF-1 are sufficient to determine the phase and amplitude of circadian rhythms *in vivo*, *ex vivo*, and *in vitro*, through increased PER synthesis. This action of insulin and IGF-1 is not restricted to any particular tissue, and facilitates circadian entrainment of gene expression and behavior to time of feeding.

Previous work suggested circadian signaling roles for metabolic cues other than insulin, such as the post-prandial glucose surge (Stephan and Davidson, 1998) and also tissue-specific roles for hormonal signals including glucagon (Mukherji et al., 2015a, Mukherji et al., 2015b), ghrelin (Verhagen et al., 2011), and oxyntomodulin (Landgraf et al., 2015). Insulin has been implicated (Tahara et al., 2011) and dismissed (Davidson et al., 2002) as a circadian messenger of feeding time, but its mechanism and function was not established and considered only for specific tissues (Balsalobre et al., 2000a, Sato et al., 2014, Yamajuku et al., 2012). Partial redundancy between insulin and IGF-1, their broad distribution, poor viability of hormone or receptor null mice (Kadowaki, 2000), and that both factors are present in sera, likely explains why core circadian signaling function was not previously ascribed to this pathway. Similarly, functional redundancy between PER paralogs and the arrhythmic phenotype of triply homozygous *Per1/2/3* null mice (Bae and Weaver, 2007) has likely obscured their transduction of feeding cues to the molecular clockwork. We do not exclude that changes in activity of other proteins or hormones may contribute to food entrainment (Delezie et al., 2016, Mukherji et al., 2015b, Pendergast et al., 2012); for example, REV-ERBα has also been implicated (Delezie et al., 2016, Mukherji et al., 2015b). However, PER induction by insulin and IGF-1 signaling is sufficient to explain how feeding cues synchronize biological clocks throughout the body.

The effect of chronic high insulin on circadian period in cells (Zhang et al., 2009) is unlikely to be physiologically relevant, as sustained hyperinsulinemia is pathological *in vivo*. In contrast, acute PER induction and resultant phase shifts by insulin are of direct physiological relevance, suggesting a general mechanism for entrainment to feeding at cellular and organismal scales. In this model, feeding triggers increased circulating insulin and free IGF-1 which, acting via increased PIP_3_, mTORC1 activation and miRNA downregulation, rapidly stimulates PER translation, sustained by increased *Per* mRNA (Figure 6A). Changes in PER protein activity are necessary and sufficient to reset the phase of circadian rhythms *in vitro* and *in vivo* (Chen et al., 2009, D’Alessandro et al., 2015), thus the magnitude and direction of phase shift elicited by insulin and IGF-1 depends on the level of PER induction, in turn dependent on prior phase, strength of stimulus, and IR and IGF-1R activity in different cells. Feeding when PER levels are already high amplifies PER oscillations, without shifting phase, to increase the number of genomic loci recruited to daily “clock-controlled” transcriptional programs.

We provide fresh insight into synchronization of cellular clocks with feeding that is consistent with current understanding (Riede et al., 2017, Mukherji et al., 2015a, Mukherji et al., 2015b) and suggest a new paradigm for entrainment of locomotor activity to feeding cycles that does not invoke an elusive “food entrainable oscillator.” Instead, because all neurons express insulin and IGF-1 receptors as well as their own circadian molecular clockwork, and as insulin and IGF-1 readily cross the blood brain barrier (Yu et al., 2006), we suggest the organization of locomotor activity with respect to feeding is functionally distributed among many brain regions that are competent to drive changes in behavior. This is consistent with lesion studies, which have failed to identify a neuroanatomical locus essential for the circadian organization of activity cycles with respect to feeding (Pendergast and Yamazaki, 2018).

Our findings also suggest a mechanistic basis for the effects of shift work on circadian rhythms. For peripheral cells of normally adapted individuals, early morning SCN-stimulated increases in glucocorticoid signaling synergize with cell-autonomous mechanisms to stimulate *Per* transcription, with PER synthesis rates then being amplified by daytime feeding via insulin and IGF-1 signaling. Through feedback repression of the PER/CRY complex upon *Per/Cry* transcription, daily PER activity regulates the timing and amplitude of *Per/Cry* expression during the next cycle; hence mistimed feeding, as during shift work, disrupts organization of circadian gene expression cycles by inappropriately timed stimulation of PER synthesis. This results in damped daily gene expression cycles (Archer et al., 2014, Dijk et al., 2012) associated with increased weight gain and susceptibility to age-linked diseases (Salgado-Delgado et al., 2013, Scheer et al., 2009). Thus, the temporal relationship between hormonal cues, particularly glucocorticoids and insulin and IGF-1, may be a key physiological dysregulation underlying the association between shift-work and ill health. Our studies indicate that this relationship may be a useful diagnostic for a “healthy body clock” and reinforce the view that management of meal timing and light exposure can help manage the adverse physiological consequences of shift-work. Additionally, interventions that maintain or reinforce the temporal organization of endocrine signaling may help to realize the societal goal of healthy aging.

## STAR★Methods

### Key Resources Table

**Table:** 

REAGENT or RESOURCE	SOURCE	IDENTIFIER
**Antibodies**		

Guinea Pig anti-CRY1	Katja Lamia, Lamia et al., 2011	N/A
Guinea Pig anti-CRY2	Katja Lamia, Lamia et al., 2011	N/A
Rabbit anti-PER1	Abcam	Cat# Ab136451
Rabbit anti-PER2	Abcam	Cat# Ab180655; RRID: AB_2630357
Rabbit anti-PER3	Abcam	Cat# Ab177482
Rabbit anti-IR	Robert Semple	N/A
Mouse anti-IGF-1	R and D Systems	Cat# MAB391; RRID: AB_2122409
Rabbit anti-AVP	Imunostar	Cat# 24286
Anti-rabbit HRP-conjugated secondary	Sigma	Cat# A6154; RRID: 258284
Anti-Guinea Pig HRP-conjugated secondary	Santa Cruz Biotechnology	Cat# Sc-2438; RRID: AB_650492
Anti-Rat HRP-conjugated secondary	Invitrogen	Cat# 629520; RRID: AB_2533965
Anti-mouse HRP-conjugated secondary	Sigma	Cat# A4416; RRID: AB_258167
Goat IgG	Santa Cuz Biotechnology	Cat# Sc-2028; RRID AB_737167
Goat anti-BMAL1	Santa Cruz Biotechnology	Cat# Sc-8550; RRID: AB_2227522
Rabbit anti-S6K	Cell Signaling	Cat# 2708; RRID: AB_390722
Mouse anti-pS6K	Cell Signaling	CAt# 9205s; RRID: AB_330945
Rat anti-tubulin	Made in-house	N/A

**Chemicals, Peptides, and Recombinant Proteins**		

insulin, recombinant	Sigma	Cat# 91077C
corticosterone	Sigma	Cat# C2505
LY294002	Sigma	Cat# L9908
ZSTK474	Selleckchem	Cat# S1072
UO126	Sigma	Cat# 19-147 EMD Millipore
rapamycin	Sigma	Cat# 553210 EMD Millipore
torin 1	Selleckchem	Cast# S2827
MHY1485	Madeleine Lancaster, Deng et al., 2015	N/A
alpha-amanitin oleate	Sigma	Cat# A7975
cycloheximide	Sigma	Cat# C7698
(R)-MG132	Sigma	Cat# M8699
BMS754807	Astra-Zeneca	N/A
D-luciferin	Biosynth	Cat# CAS [223920-67]
TTX citrate	Sigma	Cat# T5651

**Critical Commercial Assays**		

RNeasy mini kit	QIAGEN	Cat# 74104
miRNeasy mini kit	QIAGEN	Cat# 217004
Neural Tissue Dissociation Kit – Postnatal Neurons	Miltenyi Biotec	Cat# 130-094-802
Neonatal Heart Dissociation Kit – mouse and rat	Miltenyi Bioytec	Cat# 130-098-373
KAPA SYBR Fast	Sigma	Cat# SFUKB ROCHE
iScript cDNA synthesis kit	BioRad	Cat# 1708891
miRCURY LNA microRNA Inhibitor mmu-miR-29a-3p	Exiqon	Cat# YI04100172
miRCURY LNA microRNA Power Inhibitor mmu-miR-24-3p	Exiqon	Cat# YI04101706
miRCURY LNA microRNA Power Inhibitor mmu-miR30a-5p	Exiqon	Cat# YI04101046
Mm_miR-29a_1 miScript Primer Assay	QIAGEN	Cat# MS00001372
Mm_mIR-24_1 miScript Primer Assay	QIAGEN	Cat# MS00005922
Mm_miR-30a_1 miScript Primer Assay	QIAGEN	Cat# MS00011704
AKT2 Hu Radiometric Filtration Assay	MRC PPU International Centre for Kinase Profiling	N/A
AMPK Rat Radiometric Filtration Assay	MRC PPU International Centre for Kinase Profiling	N/A
Aurora B Human Radiometric Filtration Assay	MRC PPU International Centre for Kinase Profiling	N/A
BTK Human Radiometric Filtration Assay	MRC PPU International Centre for Kinase Profiling	N/A
CamK1d Human Radiometric Filtration Assay	MRC PPU International Centre for Kinase Profiling	N/A
CAMKK2 Human Radiometric Filtration Assay	MRC PPU International Centre for Kinase Profiling	N/A
CDK2 Human Radiometric Filtration Assay	MRC PPU International Centre for Kinase Profiling	N/A
CHK2 Human Radiometric Filtration Assay	MRC PPU International Centre for Kinase Profiling	N/A
CSK Human Radiometric Filtration Assay	MRC PPU International Centre for Kinase Profiling	N/A
CK1δ Human Radiometric Filtration Assay	MRC PPU International Centre for Kinase Profiling	N/A
CK2α1 Human Radiometric Filtration Assay	MRC PPU International Centre for Kinase Profiling	N/A
DYRK1a Human Radiometric Filtration Assay	MRC PPU International Centre for Kinase Profiling	N/A
DYRK3 Human Radiometric Filtration Assay	MRC PPU International Centre for Kinase Profiling	N/A
EF2K Human Radiometric Filtration Assay	MRC PPU International Centre for Kinase Profiling	N/A
eIF2AK3 Human Radiometric Filtration Assay	MRC PPU International Centre for Kinase Profiling	N/A
EPHA2 Human Radiometric Filtration Assay	MRC PPU International Centre for Kinase Profiling	N/A
EPHB3 Radiometric Filtration Assay	MRC PPU International Centre for Kinase Profiling	N/A
FGFR1 Human Radiometric Filtration Assay	MRC PPU International Centre for Kinase Profiling	N/A
VEGFR1/FLT1 Human Radiometric Filtration Assay	MRC PPU International Centre for Kinase Profiling	N/A
GSK3β Human Radiometric Filtration Assay	MRC PPU International Centre for Kinase Profiling	N/A
HIPK2 Human Radiometric Filtration Assay	MRC PPU International Centre for Kinase Profiling	N/A
IGF1R Human Radiometric Filtration Assay	MRC PPU International Centre for Kinase Profiling	N/A
IKKβ Human Radiometric Filtration Assay	MRC PPU International Centre for Kinase Profiling	N/A
IKKε Human Radiometric Filtration Assay	MRC PPU International Centre for Kinase Profiling	N/A
InsR Radiometric Filtration Assay	MRC PPU International Centre for Kinase Profiling	N/A
IRAK4 Human Radiometric Filtration Assay	MRC PPU International Centre for Kinase Profiling	N/A
JAK2 Human Radiometric Filtration Assay	MRC PPU International Centre for Kinase Profiling	N/A
JNK1/MAPK8 Human Radiometric Filtration Assay	MRC PPU International Centre for Kinase Profiling	N/A
LCK Mouse Radiometric Filtration Assay	MRC PPU International Centre for Kinase Profiling	N/A
MEK1/MAP2K1 Human Radiometric Filtration Assay	MRC PPU International Centre for Kinase Profiling	N/A
MAP3K1 Human Radiometric Filtration Assay	MRC PPU International Centre for Kinase Profiling	N/A
MAP3K11 Human Radiometric Filtration Assay	MRC PPU International Centre for Kinase Profiling	N/A
MAP3K7 Human Radiometric Filtration Assay	MRC PPU International Centre for Kinase Profiling	N/A
MAP4K2 Human Radiometric Filtration Assay	MRC PPU International Centre for Kinase Profiling	N/A
MAPK1 Human Radiometric Filtration Assay	MRC PPU International Centre for Kinase Profiling	N/A
MAPK13 Human Radiometric Filtration Assay	MRC PPU International Centre for Kinase Profiling	N/A
MAPK9 Human Radiometric Filtration Assay	MRC PPU International Centre for Kinase Profiling	N/A
MAPKAPK1b/RSK2 Human Radiometric Filtration Assay	MRC PPU International Centre for Kinase Profiling	N/A
MAPKAPK2 Human Radiometric Filtration Assay	MRC PPU International Centre for Kinase Profiling	N/A
PRAK/MAPKAPK5 Human Radiometric Filtration Assay	MRC PPU International Centre for Kinase Profiling	N/A
MARK2 Human Radiometric Filtration Assay	MRC PPU International Centre for Kinase Profiling	N/A
MARK3 Human Radiometric Filtration Assay	MRC PPU International Centre for Kinase Profiling	N/A
MARK4 Human Radiometric Filtration Assay	MRC PPU International Centre for Kinase Profiling	N/A
MELK Human Radiometric Filtration Assay	MRC PPU International Centre for Kinase Profiling	N/A
MNK1 Human Radiometric Filtration Assay	MRC PPU International Centre for Kinase Profiling	N/A
MSK1 Human Radiometric Filtration Assay	MRC PPU International Centre for Kinase Profiling	N/A
STK3/MST2/STE20 Human Radiometric Filtration Assay	MRC PPU International Centre for Kinase Profiling	N/A
NEK2 Human Radiometric Filtration Assay	MRC PPU International Centre for Kinase Profiling	N/A
NEK6 Human Radiometric Filtration Assay	MRC PPU International Centre for Kinase Profiling	N/A
p38α MAPK Human Radiometric Filtration Assay	MRC PPU International Centre for Kinase Profiling	N/A
p38β MAPK Human Radiometric Filtration Assay	MRC PPU International Centre for Kinase Profiling	N/A
P70S6K Human Radiometric Filtration Assay	MRC PPU International Centre for Kinase Profiling	N/A
PAK4 Human Radiometric Filtration Assay	MRC PPU International Centre for Kinase Profiling	N/A
PBK Human Radiometric Filtration Assay	MRC PPU International Centre for Kinase Profiling	N/A
PDK1 Human Radiometric Filtration Assay	MRC PPU International Centre for Kinase Profiling	N/A
PIM3 Human Radiometric Filtration Assay	MRC PPU International Centre for Kinase Profiling	N/A
PKAα Human Radiometric Filtration Assay	MRC PPU International Centre for Kinase Profiling	N/A
PKCα Human Radiometric Filtration Assay	MRC PPU International Centre for Kinase Profiling	N/A
PKCζ Human Radiometric Filtration Assay	MRC PPU International Centre for Kinase Profiling	N/A
PKD1 Human Radiometric Filtration Assay	MRC PPU International Centre for Kinase Profiling	N/A
PLK Human Radiometric Filtration Assay	MRC PPU International Centre for Kinase Profiling	N/A
PKCRK2 Human Radiometric Filtration Assay	MRC PPU International Centre for Kinase Profiling	N/A
RIPK2 Human Radiometric Filtration Assay	MRC PPU International Centre for Kinase Profiling	N/A
ROCK2 Rat Radiometric Filtration Assay	MRC PPU International Centre for Kinase Profiling	N/A
SGK1 Human Radiometric Filtration Assay	MRC PPU International Centre for Kinase Profiling	N/A
smMLCK Human Radiometric Filtration Assay	MRC PPU International Centre for Kinase Profiling	N/A
Src Human Radiometric Filtration Assay	MRC PPU International Centre for Kinase Profiling	N/A
SRPK1 Human Radiometric Filtration Assay	MRC PPU International Centre for Kinase Profiling	N/A
STK11 Human Radiometric Filtration Assay	MRC PPU International Centre for Kinase Profiling	N/A
STK33 Human Radiometric Filtration Assay	MRC PPU International Centre for Kinase Profiling	N/A
SYK Human Radiometric Filtration Assay	MRC PPU International Centre for Kinase Profiling	N/A
TAO kinase 1 Human Radiometric Filtration Assay	MRC PPU International Centre for Kinase Profiling	N/A
TBK1 Human Radiometric Filtration Assay	MRC PPU International Centre for Kinase Profiling	N/A
TLK1 Human Radiometric Filtration Assay	MRC PPU International Centre for Kinase Profiling	N/A
TTK Human Radiometric Filtration Assay	MRC PPU International Centre for Kinase Profiling	N/A
YES1 Human Radiometric Filtration Assay	MRC PPU International Centre for Kinase Profiling	N/A

**Experimental Models: Cell Lines**		

SV40:LUC mouse fibroblasts	Feeney et al., 2016	N/A

**Experimental Models: Organisms/Strains**		

C57BL/6 Mice	Jackson Labs	Stock no.: 00664
Cry1:LUC mouse	Maywood et al., 2013	N/A
PERIOD2::LUCIFERASE mouse	Yoo et al., 2004	MGI Cat# 3042019, RRID:MGI:3042019

**Oligonucleotides**		

Primer: Per2 Forward: CCTACAGCATGGAGCAGGTTGA	This paper	N/A
Primer: Per2 Reverse: TTCCCAGAAACCAGGGACACA	This paper	N/A
Primer: Rns18 Forward: CGCCGCTAGAGGTGAAATTC	This paper	N/A
Primer: Rns18 Forward: TTGGCAA ATGCTTTCGCTC	This paper	N/A

**Software and Algorithms**		

Prism version 7.0	Graphpad Software	N/A
BioDare	Zielinski et al., 2014https://www.biodare.ed.ac.uk	N/A
Clock Lab	Actimetrics	N/A

**Other**		

RT biolumicorder	LESA-TECHNOLOGY	N/A
ALLIGATOR	Cairn	N/A
Lumicycle^®^ 32	Actimetrics	N/A
Packard Harvester	PerkinElmer	N/A
Topcount NXT scintillation counter	PerkinElmer	N/A
P81 Unifilter plates	Merck	Cat# 7700-0512
LV200 Luminoview	Olympus	N/A
μ-Slide I 0.6 Luer	Ibidi	Cat# 80186
M119	Invitrogen	Cat# 31150-022
Corticosterone and insulin-free NS21	Made in-house, Chen et al., 2008a	N/A

### Contact for Reagent and Resource Sharing

Further information and requests for resources and reagents should be directed to and will be fulfilled by lead contact, John S. O’Neill (oneillj@mrc-lmb.cam.ac.uk).

### Experimental Model and Subject Details

#### Animals

PER2::LUC mice were originally supplied by Joe Takahashi (University of Texas Southwestern) and subsequently bred locally (University of Manchester and University of Cambridge) in a specified pathogen free barrier facility. C57/B6 mice were obtained from Charles River. For husbandry and non-experimental housing, mice were group housed with environmental enrichment under 12:12 light:dark cycles with lights on at 7am. All animal experiments were licensed under 1986 Home Office Animal Procedures Act (UK) and carried out in accordance with local animal welfare committee guidelines.

#### Organotypic slices

For organotypic slice culture, male and female PER2::LUC mice were euthanized by cervical dislocation and confirmed by exsanguination. Brain and other tissues were removed from pups (P9-10) and adult mice, respectively. SCN slices were sectioned to 300 μm thickness in ice-cold dissection medium (Gey’s balanced salt solution supplemented with 5 mg/mL glucose, 100 nM MK-801 (Sigma), 3 mM MgCl_2_, 50 μM D-APV (Sigma)) using a McIlwain “Tissue Chopper.” SCN slices were further microdissected and cultured on a Millicell membrane insert (MilliporeSigma) at 37°C, 5% CO_2_ in culture media (50% Eagle’s basal medium, 25% Earle’s balanced salt solution, 25% heat inactivated horse serum (GIBCO), 5 mg/mL glucose, 1% glutamax, penicillin/streptomycin (pen/strep), pH 7.2, 320 mOsm) for a minimum of 7 days before recording, as previously described (Hastings et al., 2005). All other tissue slices were dissected in ice-cold PBS, sectioned to 300 μm slices, and cultured and immediately recorded in 1.2 mL low glucose (5.5 mM) DMEM (D5921), supplemented with 1% glutamax, 2% (corticosterone-free and insulin-free) NS21, pen/strep and 1mM firefly luciferase.

#### Intestinal organoids

Proximal small intestinal organoids from PER2::LUC mice were established according to protocol (Sato et al., 2009). Briefly, proximal small intestine (approximately 4 cm) was isolated from male and female mice and flushed with PBSO (PBS without Mg^2+^ and Ca^2+^) until clean. Intestine was opened over the length of the organ and villi were removed by scraping with a haemacytometer coverslip. The tissue was cut in 1 cm pieces, washed by vigorous shaking in PBSO at least 3 times, and incubated in 2.5 mM EDTA in PBSO for 30-60 min while rocking at 4°C. Supernatant was checked for presence of crypts; if no crypts were present the tissue was vigorously shaken and incubated for longer. Crypts were filtered over a 70 μM filter (BD Falcon), spun down at 1200 rpm for 5 min (4°C), and subsequently washed in PBS and advanced DMEM/F12 (DMEM/F12 supplemented with 10 mM HEPES, 1x pen/strep and 1x glutamax, hereafter called Adv+++), after which crypts were spun at 600 rpm for 2 min to remove single cells. Crypts were resuspended in 25 μL Adv+++ and then taken up in basement membrane matrix (BME 2, trevigen) after which they were seeded in 10 μL drops in pre-warmed 35 mm tissue culture dishes. When matrix was solidified, expansion medium (Adv+++ supplemented with 50 ng/mL mEGF (Invitrogen Biosource), 0.5% Noggin (UPE), 0.5% R-Spondin (UPE), B27 and 12.5 mM NAC)) was added. Crypts were grown to full-grown organoids at 37°C in approximately 10-14 days, with medium replaced every 4-5 days, and were split 1 to 4-6 by mechanical disruption every 6-8 days from passage 1 onward.

#### Isolation of mouse lung fibroblasts

Mouse fibroblasts were obtained from lung tissue of adult male and female PER2::LUC mice and Cry1:LUC mice and immortalized by serial passage as for Seluanov et al. (2010). For this, mice were euthanized by cervical dislocation and confirmed by exsanguination. Lung tissue was taken and stored in ice-cold PBS. Tissue samples were subsequently removed from PBS and cut in to ∼1 mm^3^ sections using a pair of sterile scalpels, before being transferred to a 50 mL falcon tube with 10 mL “digestion medium” (DMEM/F12 supplemented with pen/strep, Mycozap Plus PR and 0.14 U/mL Liberase) and incubated at 37°C, stirring slowly, for 30 min, or until the tissue fragments turned white. The tissue fragments were then titurated using a 10 mL pipette and 40 mL “initial culture medium” (DMEM/F12, supplemented with pen/strep, Mycozap Plus PR and 15% HyClone FetalClone III) added before the tube was centrifuged at 700x g for 5 min. The resulting supernatant was discarded, the pellet resuspended in a further 20 mL “initial culture media” and the tube centrifuged for a further 5 min. The supernatant was again discarded, the pellet resuspended in 10mL “initial culture media” and transferred to a 10 cm tissue culture dish and incubated at 37°C, 5% CO_2_, 3% O_2_. After 7 days, media was refreshed and after a further 7 days, cells were split and re-plated in “selection medium” (MEM supplemented with pen/strep, non-essential animo acids, sodium pyruvate and 10% HyClone FetalClone III). After a further 2 weeks, cells were transferred to DMEM-based culture medium (DMEM supplemented with pen/strep and 10% HyClone FetalClone III). Immortalization was achieved by serial passage of cells at 37°C, 5% CO_2_, 20% O_2_. Cell lines were authenticated by observation of morphology and by continued expression of the bioluminescent reporter.

#### Primary dissociated neuron cultures

Primary cortical neuron cultures were obtained from both male and female PER2::LUC mice at birth (P0). For this, mice were euthanized by cervical dislocation and confirmed by exsanguination. Cortical tissue was dissected in a solution of EBSS without Mg^2+^ and Ca^2+^ (Invitrogen), 100 mM HEPES and pen/strep. Cells were then digested using enzymes and buffers provided in the MACS Neural Tissue Dissociation Kit – Postnatal Neurons (Miltenyi Biotec), following the manufacturers instructions. Cells were incubated at 37°C for 15 min with 650 μL enzyme mix 1 per pup. Cells were then titurated x6 using a fire-polished glass pipette. Cells were then incubated for a further 10 min at 37°C with 10 μL enzyme mix 2 per pup. An additional 5 μL enzyme mix 2 was added per pup and cells were titurated again. The entire mixture was then passed through a 50 μm filter and EBSS + 0.5% BSA (3.33 mL per pup) was added immediately before centrifugation for 5 min at 300x g. Cells were resuspended in MEM, supplemented with 20 mM glucose, 1 mM NaPyruvate, 25 mM HEPES, 1x N2 (ThermoFisher), 10% horse Serum and penicillin-streptomycin to a density of 2x10^6^ cells/mL, and 250 μL per well was seeded in PEI Borate-coated 24 well plates. Plates were incubated for 12 h and then an additional 250 μL Neurobasal A Medium, supplemented with 2% B27, 1% glutamax and pen/strep was added per well. Cells were maintained at 37°C, 5% CO_2._ Cells subsequently underwent a half media change every week for 3 weeks before experimentation.

#### Isolation of primary cardiomyocytes

Primary cardiomyocytes were isolated from both male and female PER2::LUC neonatal pups (P2-P4) using the heart dissociation kit (Miltenyi Biotec) as per manufacturer’s instructions. Briefly, pups (p3-4) were euthanized by cervical dislocation, heart tissue removed and store on ice-cold ADS (106 mM NaCl, 20 mM HEPES, 0.8 mM NaH_2_PO_4_, 5.3 mM KCl, 0.4 mM MgS0_4_, 5 mM glucose). Vessels and connective tissue were removed and the resulting cardiac tissue placed in fresh ice-cold ADS. Tissue was further dissected in to 1mm^3^ sections and placed in a 50 mL tube. Tissue sections were allowed to settle, supernatant removed, 2.5 mL of ‘enzyme mix’ added and the tissue incubated at 37°C for 30 mins. Tissue was then titurated 5 times using a 5 mL tissue culture pipette, before incubating for further 30 min and titurating a second time. 7.5 mL of medium 1 (DMEM high glucose supplemented with 17% M199, 10% horse serum, 5% new born calf serum, Glutamax, Pen/Strep, and Mycozap) was added, the tissue suspension filtered through a 70 μm strainer and centrifuged for 5 min at 600x g with no brake. The pellet was resuspended in medium 1 and plated in 10 cm Petri dishes to remove fibroblasts via negative selection. After 2 h of incubation at 37°C the supernatant was collected and spun down for 5 min at 600x g. The pellet was resuspended in medium 1, cells were counted and seeded in a 96-well plate pre-coated with 10ug/mL fibronectin (100,000 cells per well). The day after isolation the medium was changed and cardiomyocytes were cultured in medium 2 (DMEM high glucose supplemented with 17% M199, 5% horse serum, 0.5% new born calf serum, glutamax, pen/strep, and mycozap) at 37°C, 5% CO_2_ for 7 further days before being used experimentally.

### Method Details

#### Experimental cell culture

Cells were grown to confluence (passage no. < 40) in experimental dishes in high-glucose (27.8 mM), glutamax-containing DMEM (GIBCO) supplemented with 10% serum (HyClone FetalClone III, Themofisher) and pen/strep. Confluent cultures were kept for up to 4 weeks with media refreshed every 5-7 days. Before the start of recording, unless otherwise described, cells were synchronized by external temperature cycles of 12 h 32°C followed by 12 h 37°C for a minimum of 72 h, then changed to serum-free, B27-free “Air Media” (Bicarbonate-free, DMEM, 5mg/mL glucose, 0.35 mg/mL sodium bicarbonate, 0.01 M HEPES, 2 μg/mL pen/strep, 1% Glutamax, 1 mM luciferin, pH 7.4, 350 mOsm; adapted from Hastings et al., 2005), and dishes sealed. For experiments in the absence of extracellular glucose, glucose was excluded from the stock medium. Cells were then transferred to a Lumicycle^®^ luminometer where bioluminescent activity was recorded at 10 min intervals, or an ALLIGATOR (Cairn Research), where bioluminescent activity was recorded at 15 min intervals using an electron multiplying charge-coupled device (EM-CCD) at constant 37°C.

#### Bioluminescence from tissue slices

For bioluminescent recording, SCN slices were transferred into 1.2 mL modified air medium. This was made as previously described (Hastings et al., 2005) and modified to be serum-free, corticosterone-free, and insulin-free NS21 (Bicarbonate-free DMEM, 5 mg/mL glucose, 0.35 mg/mL sodium bicarbonate, 0.01M HEPES, 2 μg/mL pen/strep, 1% glutamax, 6% corticosterone and insulin-free NS21, 0.3 mM d-luciferin). All other tissue slices were cultured and recorded in 1.2 mL low glucose (5.5 mM) DMEM (D5921), supplemented with 1% glutamax, 2% (corticosterone-free and insulin-free) NS21 and 1 mM d-luciferin. Slices were placed in a 35mm polystyrene Petri dish and sealed with a coverslip and vacuum grease. SCN slices were treated with 1 μM TTX citrate and 600 nM insulin. Whole SCN slice bioluminescence was recorded using Hamamatsu photomultiplier tube assemblies housed within a light-tight 37°C incubator, while whole tissue slices were imaged using an ALLIGATOR. Time-lapse imaging of bioluminescent SCN slices was performed using an LV200 Luminoview (Olympus). Bioluminescent images were acquired over a 30 min interval using a C9100-13 EM-CCD camera (Hamamatsu).

#### Bioluminescence from organoids

For recordings, 60-80 μL crypts were seeded in 35 mm dishes and grown in expansion medium supplemented with 1 mM d-luciferin. After three days, cells were changed into “Air Media” (Bicarbonate-free DMEM, 5 mg/mL glucose, 0.35 mg/mL sodium bicarbonate, 0.01 M HEPES, 2 μg/mL Pen/Strep) supplemented with 0.5% Noggin, 0.5% R-spondin, 12.5 mM NAC, and 1 mM luciferin. Luciferase activity was assayed in a Lumicycle-32 (Actimetrics). Water or insulin (1 μg/mL) was added on a 37°C heat pad after 48 h of recording, after which recording was continued.

#### Insulin and drug treatment

All drugs were added prior to insulin treatment and given 5-30 min to equilibrate at 37°C (unless otherwise stated) before insulin was added. Drug concentrations were determined empirically and were, unless otherwise stated, used at the following final concentrations: LY294002 – 100 μM, ZSTK474 - 10 μM, UO126 – 4 μM, rapamycin – 400 nM, Torin 1 - 1 μM, MHY1485 – 2 μM, alpha-amanitin oleate – 100 nM, cyclohexamide – 50 μM, MG132 – 10 μM, insulin – 600 nM, EGF – 20ng/mL, FGF – 100 ng/mL, glucose – 11 mM, BMS754807 – 1 μM, PF670462 – 3 μM, TTX citrate - 1 μM, VO-OHpic – 100 nM, 4EGI-1 - 100 μM, forskolin - 1 μM, IBMX - 100 μM. Insulin was dissolved in dilute HCl to form a 10 mg/mL stock solution and stored at −20°C in aliquots. Before use, this was further diluted to 1 mg/mL in cell culture media and added to cells to give a final concentration of 600 nM, unless otherwise stated. Cells and slices were maintained at 37°C for the duration of the treatment through use of an isothermal pad. Except when performed under perfusion, drugs and insulin remained in the media from their addition for the duration of the experiment.

#### Perfusion cell culture and experimentation

For experiments involving application of an acute bolus, recordings were carried out under perfused media conditions, as for Crosby et al. (2017). Cells were seeded and grown to confluence in a microfluidics slide (μ-Slide I 0.6 Luer, Ibidi) in high glucose (27.8 mM), glutamax-containing DMEM (GIBCO) with 10% serum (HyClone FetalClone III) and pen/strep. Cultures were incubated for 1-2 weeks, with DMEM media refreshed every 5-7 days. Cells were also subjected to temperature entrainment cycles during this time. Before the start of recording, cells were changed to low-glucose (5.5 mM) DMEM (Sigma) supplemented with 1% glutamax, pen/strep and 1 mM luciferin. Cells were then perfused with the same media, driven by syringe pump (NE-1600) at a flow rate of 50 μl/hr for the duration of the experiment. Recording was performed using an ALLIGATOR (Cairn Research). Boli were applied using a diversion of flow, with no change in flow rate or detectable flow disturbance.

#### Immunoprecipitation

For immunoprecipitation, cells were synchronized by temperature cycles and harvested by scraping on ice in cold PBS and centrifuged at 100x g at 4°C for 5 mins. The resulting pellet was resuspended in 150 μL lysis buffer (50 mM tris, pH 7.5, 1% Triton X-100, 1.5 mM MgCl_2_, 100 mM NaCl, 100 mM MgCl_2_, 100 U/μl DNase, cOmplete protease inhibitor cocktail (Roche)) and incubated on ice for 10 min. 750 μl wash buffer (50 mM Tris, pH 7.5, 1% Triton X-100, 1.5 mM MgCl_2_, 5 mM EDTA, 100 mM NaCl) was then added and the sample centrifuged at max speed at 4°C for 15 mins. 75 μl of the resulting supernatant was removed for use as a control for total lysate. The result of the sample was incubated with 30 μl antibody-bound agarose beads for 2 h at 4°C with constant agitation. Beads were then washed three times in wash buffer before the beads were resuspended in 1x sample buffer and heated to 70°C for 10 min. Samples were then loaded on to gels for electrophoresis using a fine insulin needle.

#### Gel Electrophoresis and Western Blotting

We used NuPAGE Novex 4%–12% Bis-Tris gradient gels (Life Technologies), and ran them using the manufacturer’s protocol with a reducing MES SDS buffer system. Protein transfer to nitrocellulose for blotting was performed using the iBlot system (Life Technologies), with a standard (P0, 8 mins) protocol. Nitrocellulose was washed briefly, and then blocked for 30 mins in 5% w/w non-fat dried milk (Marvel) in Tris buffered saline/0.05% Tween-20 (TBST). Membranes were then incubated in antibody diluted in blocking buffer (5% milk, TBST) overnight at 4°C. The following day, membranes were washed for 10 min three times (in TBST) and then incubated with 1:10,000 HRP-conjugated secondary antibody (Sigma-Aldrich) diluted in blocking buffer for 60 min. Three more 10 min TBST washes were then performed before chemiluminescence detection using Immobilon reagent (Millipore) and imaging using a Gel-Doc XR^+^ system (Bio-Rad). Quantification was performed using Image Lab Software 6.0 (Bio-Rad).

#### Quantitative PCR for mRNA

For qPCR, cells were grown to confluence in 35mm dishes, synchronized by temperature cycles and changed in to serum and B27-free “Air Media” as described above. After treatment for the time indicated, cells were harvested and RNA was extracted using the RNeasy mini kit (QIAGEN) using the recommended protocol. On column DNase digestion was performed. cDNA was generated from these samples using the iScript cDNA synthesis kit (BioRad) using a total RNA concentration of 100 ng per sample. Samples were then kept on ice while they were made up to a volume of 100 μL with nuclease-free water. Small quantities of all samples were pooled and subsequently serially diluted to make standards. Samples were diluted ten-fold to ensure their values fell within the standard curve. qPCR was carried out using a Prime Pro 48 machine (Techne) and KAPA SYBR Fast qPCR reagents (KAPA Biosystems) as per manufacturers instructions. The primer sequences used are described in the key resources table. All pairs of primers were annealed at 58°C, and a melt curve performed. Data was then analyzed and outliers excluded using the Prime Pro Study software. Values were normalized to the standard curve and against RNS18.

#### Quantitative PCR for miRNA

For qPCR for miRNA, cells were harvested and miRNA isolated using an RNAeasy miRNA isolation kit (QIAGEN) as per the manufacturers protocol. On column DNase digestion was not performed. cDNA was generated using the miScript II RT kit (QIAGEN). A total reaction volume of 20 μL consisted of 4 μL HiSpec Buffer, 2 μL miScript nucleics mix, 2 μL reverse transcriptase mix and 500 ng RNA, made up to volume with nuclease-free water. Samples were then incubated at 37°C for 60 mins, followed by 5 min at 95°C. Samples were kept on ice while they were made up to a volume of 100 μL with nuclease-free water. Pooled standards and further dilution of samples was performed as for qPCR for mRNA. qPCR was performed using the miScript SYBR green PCR kit. Wells consisted of 5 μL QuantiTect SYBR green PCR master mix, 1 μL miScript Universal primer, 1 μL Primer Assay and 1 ng cDNA, made up to 10 μL with nuclease-free water. The plate was then sealed, vortexed and centrifuged briefly before placing in the Prime Pro 48 qPCR machine. Wells were then subjected to 15 min at 95°C, followed by 40 cycles of 15 s 94°C, 30 s at 55°C and 30 s at 70°C. A melting step was also performed. Results were analyzed and outliers excluded using the Prime Pro Study software. All values were then normalized against RNU6.

#### Immunohistochemistry

Following careful dissection, brains were immediately post-fixed in 10 mL 4% paraformaldehyde (PFA) made up in phosphate buffer (108 mM Na_2_HPO_4_.2H_2_O, 25.3 mM NaH_2_PO_4_.2H_2_O) and shaken for 4-5 h at room temperature before being cryopreserved in 20% sucrose in PBS at 4°C overnight. The brains were then mounted on a freezing microtome using OCT embedding medium (Thermo Scientific) and 40 μm coronal sections were taken, rostral to caudal. Sections were placed in wells of WHO dimple trays containing 0.01 M PBS. Sections were washed twice in PBS and then blocked for 1 h at room temperature with shaking in 2% serum in Day 1 Buffer (0.01 M PBS, 1% BSA, 0.3% Triton-X) to reduce non-specific binding (serum donor was the same as that of the secondary antibody). Sections were then incubated in primary antisera in Day 1 Buffer overnight at 4°C with shaking. Sections were next washed in Day 2 Buffer (1:3 dilution of Day 1 Buffer in 0.01 M PBS) twice and incubated with appropriate secondary antibodies, diluted 1:500 in Day 2 Buffer for 1 h with shaking at room temperature. Sections were washed twice more in Day 2 Buffer, then in 0.01 M PBS before mounting onto Superfrost Plus slides (Thermo Fisher), rinsing in water and coverslipping using Vectashield Hardset mounting medium with DAPI.

#### Polyribosome Fractionation

Polysome fractions were prepared as for Gandin et al. (2014). Briefly, cells were grown to confluence in a 125cm^2^ flask. Immediately prior to harvesting, cells were incubated with 100 μg/mL cycloheximide for 5 min at 37°C. Cells were then placed on ice and washed twice with 10 mL ice-cold PBS containing 100 μg/mL cycloheximide. Cells were then scraped in 5 mL ice-cold PBS with 100 μg/mL cycloheximide and centrifuged at 200x g at 4°C for 5 min. The resultant pellet was resuspended in 425 μL hypotonic buffer (5 mM Tris-HCl, 2.5 mM MgCl_2_, 1.5 mM KCl, 1x protease inhibitor cocktail (Roche)). 5 μL 10 mg/mL CHX, 1 μL of 1 M DTT and 100 units of RNase inhibitor (ThermoFisher) were added and the mixture vortexed for 5 s. Triton X-100 and sodium deoxycholate were added to total 0.5% of each and vortexed again for 5 s. The lysate was then centrifuged at 16,000x g at 4°C for 7 min and the pellet discarded. The remaining supernatant was made up 750 μL using RNA-free water and added to 750 μL Trizol. 500 μL of sample was then loaded on to pre-poured sucrose gradients (5%–50% sucrose in H_2_O with 200 mM HEPES, 1 M KCl, 50 mM MgCl_2_, 100 μg/mL cycloheximide, 1x protease inhibitor cocktail and 100units/mL RNase inhibitor). The gradients were then ultracentrifuged at 222,228x g for 2 h at 4°C using a SW41Ti rotor in ‘low brake’ mode. Following centrifugation, the samples were separated in to 10 × 1 mL fractions using a fraction collector, with simultaneous detection of UV absorbance at 254 nm.

#### miRNA inhibition

Inhibition of miRNAs was performed using miRCURY LNA microRNA Inhibitors and miRCURY LNA microRNA Power Inhibitors (Exiqon). Cells were transfected using HiPerFect transfection reagent (QIAGEN). For fibroblasts, each well of a 24-well plate was filled with 100 μL transfection mix (100 μL Opti-MEM®, supplemented with 5 nM microRNA inhibitor and 3 μL HiPerFect transfection reagent). Cells in 500 μL complete medium (DMEM supplemented with 10% HyClone FetalClone III and pen/strep) were then seeded in to these wells at a density of 3x10^6^ cells per well. For cardiomyocytes, 20 μL of the above transfection mix was added per well of a 96-well plate. Plates were incubated for 20 h at 37°C. Medium was changed and the cells subjected to temperature cycles for 48 h before being changed in to experimental conditions as previously described.

#### Kinase selectivity profiling

Kinase selectivity profiling was performed through contract research services at the MRC PPU International Centre for Kinase Profiling, Dundee, UK., using their radioactive filter binding assay methodology as previously described (Hastie et al., 2006). Briefly, BMS-754807 (AstraZeneca) was tested at a single concentration of 1 μM, in duplicate, against 76 kinases. BMS-754807 was pre-incubated at room temperature for 5 min in the presence of 5-20 mU of kinase enzyme and the respective peptide/protein substrate, before initiation of the reaction by addition of ^33^P-γ-ATP. Assays were incubated at room temperature for 30 mins before termination by the addition of 50 mM orthophosphoric acid. Assay plates were harvested onto P81 Unifilter Plates (Whatmann) by a Packard Harvester (PerkinElmer) using a wash buffer of 50 mM orthophosphoric acid and subsequently dried in air. The dry Unifilter plates were then sealed on the addition of MicroScint (PerkinElmer) and counted using a Topcount NXT scintillation counter (PerkinElmer). Data was reported as a % incorporation of radiolabelled phosphate from ^33^P-γ-ATP into the peptide or protein substrate in relation to the maximal enzyme control.

#### *In vivo* IR/IGF-1R inhibition

PER2::LUC mice (male and female, 12-14 weeks of age) were individually housed, and locomotor activity monitored via wheel-running activity in light-tight temperature controlled cabinets (Actimetrics). Animals were administered with either 500 μM BMS-754807, or a vehicle control, *ad libitum* in the drinking water for the duration of the experiment. Drinking water was also supplemented with 10% concentrated sugar-free blackcurrant and apple squash (Co-operative foodstores). Mice were subjected to 12:12 LD cycles for 7 days, followed by 7 days of constant darkness. Mice were subsequently euthanized by cervical dislocation, confirmed by exsanguination.

#### Restricted feeding behavioral monitoring

PER2::LUC mice (male and female, 12-14 weeks of age) were individually housed, and locomotor activity monitored *via* wheel-running activity in light-tight temperature-controlled cabinets (Actimetrics). Drinking water was supplemented with 10% concentrated sugar-free blackcurrant and apple squash (Co-operative foodstores). Following 7 days in a 12:12 LD cycle (6am-6pm) with food available *ab libitum*, all groups were transferred to constant light (100 lux). Simultaneously, 2 groups were subjected to restricted feeding with food available for 8 h/day (8am-4pm). At this time, one of these groups was also switched to drinking water further supplemented with 500 μM BMS-754807, with all other groups receiving a vehicle control. After 10 days, all mice were returned to *ad libitum* feeding for the remainder of the experiment. Mice were subsequently euthanized by cervical dislocation, confirmed by exsanguination.

#### Locomotor Activity Onset

C57/BL6J mice (males, 10-12 weeks of age) were individually housed, and locomotor activity monitored using a beam break detection floor grid. Mice were placed into constant darkness (DD) for 7 days, after which food was removed for 36 h. Approximately 20 h into the fasting period, mice received glucose (3 g/kg)/insulin (2.5 IU/kg) or saline at CT20, and were then maintained in DD for a further 10 days.

#### *In vivo* bioluminescence with IP injection

Male and female PER2::LUC mice (9-12 weeks of age) were shaved across the abdomen and back to maximize bioluminescence detection. Mice were singly housed in LESA-biolumicorders (LESA technologies) with luciferin provided *ad libitum* in the drinking water (1 mg/mL; Promega). After 3 days of recording, mice received vehicle (saline, n = 13), insulin (2.25 IU/kg, n = 9), glucose (3 g/kg, n = 8), or both (n = 8) by intraperitoneal injection (IP) soon after the peak in PER2::LUC bioluminescence. In separate studies, mice received insulin (2.5 IU/kg or 1 IU/kg) and glucose (3 g/kg or 1.3 g/kg) or vehicle (saline) at the trough of PER2::LUC bioluminescence (n = 3-4/group). For insulin tolerance testing, circulating glucose was assessed following administration of insulin (2.5 IU/kg, IP), glucose (3 g/kg, IP) or both in separate mice. Circulating blood glucose was monitored with an Accu-chek Aviva glucose meter (Roche) at time 0, 30, 60 and 120 min post administration. Blood was sampled from the tail vein

#### Food timing reversal

For food reversal experiments, bioluminescence was monitored in singly housed male and female PER2::LUC mice (9-12 weeks of age) under constant darkness. Mice were acclimatized to the biolumicorder cages with *ad libitum* access to food and water. BMS treated mice received 500 μM BMS-754807 in the drinking water for the duration of the experiment. In preparation for the reversal in food availability, food was removed from the cages at 7am then returned at 7pm (reflecting ZT0 and ZT12 of the previous LD cycle, respectively) for an additional 2 days. Upon reversal of food availability, mice were provided with food each day at 7am. During ZT0 feeding, the amount of food available set to ∼3 g/day. Bioluminescence was monitored for a further 5 cycles under daytime food restriction.

### Quantification and Statistical Analysis

#### For mouse behavior

Blinded assessment of phase shift, acrophase and behavioral period was determined using ClockLab software (Actimetrics).

#### For *in vivo* bioluminescence

For analyses, bioluminescent recordings were normalized using a 48 h moving average, and induction following treatment calculated as the average normalized bioluminescence 0-2 h post-injection.

#### Circadian analyses

Analysis was performed using GraphPad Prism version 7.0, with the exception of bioluminescent recordings of organotypic slices of SCN, which were analyzed using BioDare (Zielinski et al., 2014). Where required for clear interpretation, data was min-max normalized. Bioluminescent traces from organotypic slices of SCN are normalized to 24 h period to allow for comparison between slices. Bioluminescence traces from cortical neurons are de-trended using a one-phase decay curve to allow for clear comparison between conditions. Bioluminescent traces of cells were fitted with damped cosine waves using the following equation:

$$y=mx+c+Amplitude\cdot e^{-kx}\cdot \text{cos}\left(\frac{2\pi \left(x-phase\right)}{period}\right)$$

where y is the signal, x the corresponding time, amplitude is the height of the peak of the waveform above the trend line, k is the decay constant (such that 1/k is the half-life), phase is the shift relative to a cos wave and the period is the time taken for a complete cycle to occur. This method allows objective quantification of period and phase, but is not useful for comparing amplitudes at discrete time points following experimental perturbations. As such, amplitude is measured as a percentage, where 100% is the difference between the peak and the trough of the vehicle control at a specified time point. Unless otherwise defined, this point is the first circadian peak in PER2 expression following treatment, excluding any acute/transient change in bioluminescence that the treatment might induce. For animal bioluminescence, where amplitude is considered as a repeated-measure, amplitude is calculated as the peak-trough in the cycle following IP injection as a percentage of the peak-trough of the cycle preceding insulin injection. Induction of PER2 is measured using ΔPER2::LUC, defined as the difference in the bioluminescent signal between the time of manipulation and the subsequent peak in bioluminescence, or the equivalent time point in the untreated control. The units used here are relative luminescence units (RLU) and are consistent within each experiment. Phase shifts were determined relative to a vehicle treated control.

#### Pixel analysis of SCN slices

SCN bioluminescence time-lapse images were processed in FIJI (Schindelin et al., 2012) by performing background subtraction, and despeckling to remove noise. Pixels within the lateral region of the SCN were then grouped as “superpixels” (4x4 – 6x6 pixels) to serve as regions of interest (ROIs) within each individual SCN slice. Intensity profiles over time were produced for each ROI in FIJI and were normalized to the minimum and maximum values for each ROI in Microsoft Excel. This data was analyzed using BioDare 2 software (Zielinski et al., 2014) to obtain period and phase values for each ROI within the slice. Phase is determined relative to the window of analysis. Period analysis was performed in Prism 7.0. To generate Rayleigh plots to visualize phase coherence within a slice, phase values were converted into radians using Microsoft Excel before plotting using the ‘circular’ package in R.

#### Statistics

Data is presented as mean ± SEM, and statistical significance of mean differences determined using Welch’s t test (t test), one-way ANOVA (OWA) and two-way ANOVA (TWA) as indicated in the figure legend, with the multiple comparisons test (MCT) used also reported in the figure legend. TWA_int_ indicates the ANOVA of interaction. The sample size (n) is also reported in the figure legend for each experiment, with n defined as the number of identically-treated replicates. Statistical analyses were performed using Graphpad Prism. P-values are reported using the following symbolic representation: ns = p > 0.05, ^∗^ = p ≤ 0.05, ^∗∗^ = p ≤ 0.01, ^∗∗∗^ = p ≤ 0.001, ^∗∗∗∗^ = p ≤ 0.0001.
